# Self-Healing Polymer-Based Coatings: Mechanisms and Applications Across Protective and Biofunctional Interfaces

**DOI:** 10.3390/polym17233154

**Published:** 2025-11-27

**Authors:** Aldo Cordoba, Fabiola A. Gutiérrez-Mejía, Gabriel Cepeda-Granados, Juan V. Cauich-Rodríguez, Karen Esquivel Escalante

**Affiliations:** 1 Graduate and Research Division, Engineering Faculty, Universidad Autónoma de Querétaro, Cerro de las Campanas, Queretaro 76010, Mexico; acordoba07@alumnos.uaq.mx (A.C.); gcepeda21@alumnos.uaq.mx (G.C.-G.); 2 Department of Quantitative Methods, Universidad Anáhuac Campus Querétaro, Circuito Universidades l, Kilómetro 7, Fracción 2, El Marqués, Queretaro 76246, Mexico; 3 Unidad de Materiales, Centro de Investigación Científica de Yucatán, C. 43 No. 130 x 32 y 34, Col. Chuburná de Hidalgo, Merida 97205, Mexico; fagutierrezmejia@gmail.com

**Keywords:** self-healing, polymers, coatings, corrosion protection, biomedical applications

## Abstract

Self-healing polymer-based coatings have emerged as a new generation of adaptive protective materials capable of restoring their structure and function after damage. This review provides a comprehensive analysis of current strategies enabling autonomous or externally triggered repair in polymeric films, including encapsulation, reversible chemistry, and microvascular network formation. Emphasis is placed on polymer–inorganic hybrid composites and vitrimeric systems, which integrate barrier protection with stimuli-responsive healing and recyclability. Comparative performance across different matrices—epoxy, polyurethane, silicone, and polyimine—is discussed in relation to corrosion protection and biomedical interfaces. The review also highlights how dynamic covalent and supramolecular interactions in hydrogels enable self-repair under physiological conditions. Recent advances demonstrate that tailoring interfacial compatibility, healing kinetics, and trigger specificity can achieve repeatable, multi-cycle recovery of both mechanical integrity and functional performance. A representative selection of published patents is also shown to illustrate recent technological advancements in the field. Finally, key challenges are identified in standardizing evaluation protocols, ensuring long-term stability, and scaling sustainable manufacturing. Collectively, these developments illustrate the growing maturity of self-healing polymer coatings as multifunctional materials bridging engineering, environmental, and biomedical applications.

## 1. Introduction

Self-healing polymer-based coatings constitute a rapidly advancing field, bridging materials science, engineering, and biomedicine. These coatings can autonomously or non-autonomously repair damage, offering enhanced durability, barrier protection, and bio functionality for diverse applications [1]. A polymeric coating can be defined as a thin layer of any polymeric material that can be used for physical or corrosion protection [2]. Conventional coatings have been traditionally used on metals, ceramics, and polymeric substrates [3]. Originally, paints were used as an organic polymeric coating for environmental protection. Typically, failure of these coatings can be due to a combination of diverse factors such as misapplication of the coating, defective coating, the wrong choice of coating, or exposure to an unanticipated environmental excursion [4,5]. In addition to this, during its service life, the coating film undergoes changes in mechanical properties, leading to the formation of microcracks, which subsequently propagate and expose the substrate to atmospheric moisture and oxygen [5]. This action results in accelerated disbonding of the paint and flake formation from the metal coating interface. Therefore, more complex and heterogeneous composite polymeric coatings are used as a barrier for moisture and ions where this is chemically bonded to the substrate [6]. In some cases, multilayer coatings are employed where a primer is first deposited for initial protection and adhesion to the substrate, then an intermediate functional layer, and finally a topcoat for environmental protection (UV, water, ions, mechanical stress) or aesthetic purposes [7]. That is why self-healing polymer-based coatings provide several key advantages over traditional polymer coatings, particularly in terms of longevity, maintenance, and protective performance [8]. Self-healing polymers can autonomously or externally repair scratches, cracks, and micro-damage, restoring protective function [9], maintain or restore high corrosion resistance after damage, even in harsh environments [10], significantly extend life service due to self-repair, reduce the need for frequent recoating [1], and manual inspection and repair [8]. Furthermore, the environmental impact has decreased because newer self-healing coatings can be waterborne, VOC-free, and recyclable [2], and some of them can be designed to respond to stimuli (light, heat, moisture) for targeted healing [11]. Despite significant advances in the field of self-healing polymers, the current literature remains fragmented, with most works addressing either extrinsic healing systems (e.g., microcapsules, nanocontainers, microvascular networks) or intrinsic dynamic polymer networks (e.g., vitrimers) in isolation. Furthermore, the performance of these systems is often discussed separately for corrosion protection, biomedical films, or structural applications, without an integrated comparison. This review aims to address this gap by critically examining how different self-healing mechanisms, polymer matrices, and dynamic chemistries influence protective performance and functional recovery in coatings, thus providing a unified perspective that highlights their strengths, limitations, and application-specific requirements.

The objective of this review is to provide a comprehensive and comparative analysis of self-healing polymer-based coatings by (i) summarizing the fundamental mechanisms governing extrinsic and intrinsic healing; (ii) critically evaluating recent advances in vitrimer chemistries within epoxy, PDMS, polyurethane, and polyimine systems; (iii) analyzing polymer–inorganic hybrid composites and microvascular strategies used in corrosion protection; and (iv) identifying performance gaps, testing challenges, and future research directions required to advance robust, multi-cycle, and application-specific self-healing coatings.

## 2. Self-Healing

**Figure 1:**
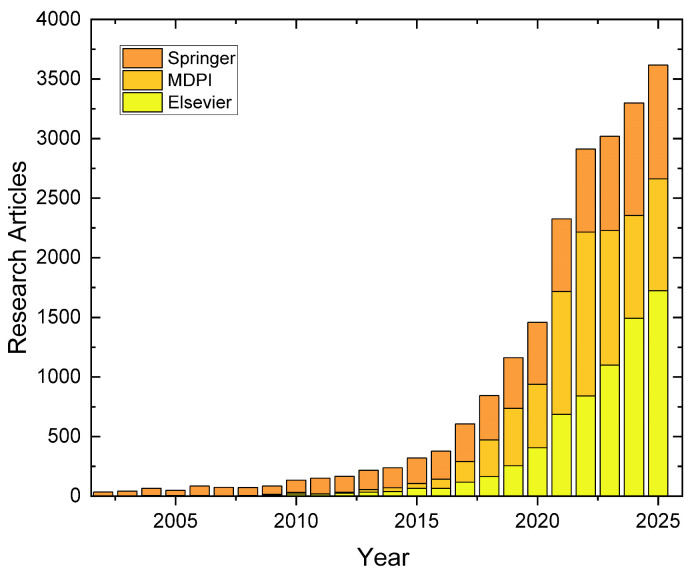
Temporal evolution of publications related to self-healing polymers.

Polymeric materials, despite their versatility, are susceptible to the accumulation of damage, such as microcracks, during their service life, which inevitably leads to a degradation of their properties and, ultimately, to catastrophic failure. In response to this fundamental challenge, a class of smart materials known as self-healing materials emerged several decades ago [12,13]. Over the past two decades until today, research on self-healing materials has expanded rapidly. As shown in Figure 1, the number of publications of three editorial houses has grown exponentially, reflecting the rising relevance and multidisciplinary nature of this field (see Figures S1 and S2).

These systems are designed with the inherent ability to repair damage autonomously or in response to an external stimulus. The concept is directly inspired by biological systems, emulating processes such as blood coagulation, skin scarring, or the consolidation of fractured bones, where damage triggers an intrinsic repair response [14,15,16]. The motivation behind this area of research is both scientific and economic. The main objective is to significantly extend the service life of materials, improve the safety and reliability of structures, reduce costs associated with maintenance and replacement, and promote sustainability by minimizing waste and the consumption of non-renewable resources. The growing industrial interest is reflected in market projections, which anticipate a considerable expansion in the application of these technologies in the coming years.

Advances in self-healing represent a fundamental paradigm shift in materials science. For centuries, material development focused on damage prevention, that is, on designing increasingly robust systems to delay the formation and propagation of defects. Self-healing, in contrast, adopts a philosophy of damage management: it accepts that damage is inevitable and, consequently, integrates a mechanism to manage and repair it in situ [12,14,15,17]. This transition from passive materials to active and “living” materials that respond to their environment has profound implications for the design of more resilient and durable structures in fields ranging from aerospace and automotive engineering to consumer electronics and biomedical applications [18,19].

The general repair process, regardless of the specific mechanism, can be conceptualized in three fundamental stages: (1) the activation or triggering of the repair response, which is initiated by the damage event itself; (2) the transport of the healing components to the damaged site; and (3) the chemical repair or remodeling process that restores the structural and functional integrity of the material [14,20].

### 2.1. Classification of Self-Healing Mechanisms

To understand the design, capabilities, and limitations of self-healing materials, it is essential to classify them according to the fundamental principles that govern their functionality. The two most important classifications are based on the origin of the healing capability (intrinsic vs. extrinsic) and the nature of the stimulus that triggers the process (autonomous vs. non-autonomous).

#### 2.1.1. Intrinsic vs. Extrinsic Mechanisms: The Origin of Healing Capability

This classification focuses on whether the ability to repair an inherent property of the polymer matrix is or if it is achieved by adding an external healing agent.

**Figure 2:**
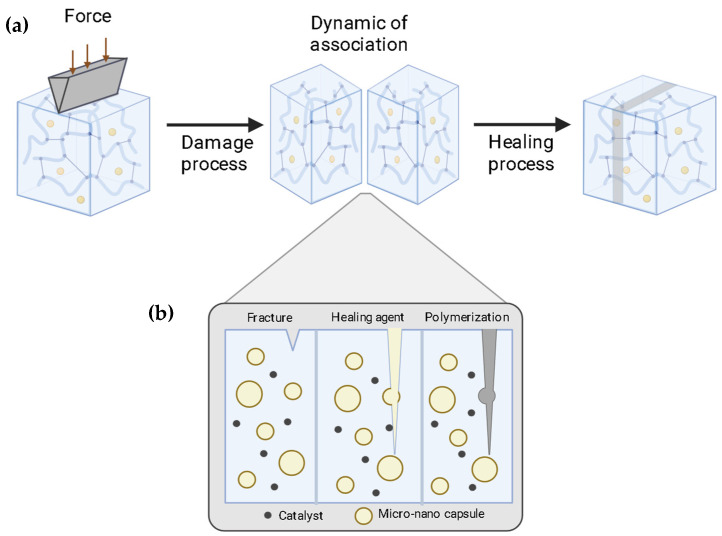
Self-healing process after crack formation (**a**) and complete rupture (**b**) using a monomer containing micro/nano capsules and a catalyst.

Extrinsic self-healing is defined as the healing functionality that is provided by a discrete, non-structural healing agent that is pre-embedded within the polymer matrix. This healing agent is isolated in containers, such as microcapsules, hollow nanofibers, or vascular networks, and is released when damage (e.g., a crack) ruptures these containers, allowing the agent to flow and repair the affected area (Figure 2). Its advantages include high and rapid healing efficiency, making it ideal for acute damage events, and its relative ease of implementation in a wide range of existing polymer systems. However, its main disadvantage is that the healing capability is finite and generally limited to a single event, as the healing agent is consumed once released. Furthermore, the presence of these containers can act as a defect within the matrix, potentially compromising the mechanical properties of the pristine material [14,16,18,21,22].

Intrinsic self-healing refers to systems where the ability to heal is an inherent property of the polymer matrix itself, derived from the presence of dynamic or reversible bonds in its molecular architecture. Repair is achieved by reforming these bonds across the damaged interface, a process that is often activated by an external stimulus such as heat or light. The most significant advantage of this approach is the potential to achieve multiple and repeated healing cycles at the same location, as the system does not rely on a finite reservoir of healing agents. However, these systems often face a fundamental trade-off between mechanical robustness and healing capability. The dynamic bonds that allow for repair are, by nature, more labile than permanent covalent bonds, which can compromise the load-bearing capacity of the material. Additionally, the healing process can be slower and require specific conditions (e.g., high temperatures) to achieve adequate molecular mobility [14,19,23].

Nevertheless, it is important to say that in the intrinsic self-healing mechanism, chemical and physical properties of the material are exploited by dynamic covalent bonds (disulfide linkages for example) or non-covalent interactions (hydrogen bonding, hydrophobic interactions and π–π stacking, electrostatic interactions) that can be reversed by heat, pH, light, ligands or biomolecules, mechanical stress, pressure, etc.

The historical evolution of the field reflects a direct response to these limitations. Initial research focused heavily on extrinsic systems due to their conceptual simplicity and high efficiency for a single repair event. However, the critical limitation of “single-use” repair became apparent, as materials in real-world applications often experience repeated damage cycles. This deficiency directly spurred the development of intrinsic systems, which, by their nature of being based on reversible chemistry, offered the promise of multiple healing cycles. Therefore, the progression from extrinsic to intrinsic is not accidental, but a logical evolution driven by the need to overcome the shortcomings of the previous generation of materials [19,24].

#### 2.1.2. Autonomous vs. Non-Autonomous Repair: The Trigger of the Process

This classification focuses on the stimulus that initiates the repair process and is orthogonal to the previous classification. Autonomous systems are characterized by their ability to initiate the repair process without any external intervention; the damage event itself acts as the sole trigger. This is the hallmark of most extrinsic systems, where the propagation of a crack mechanically ruptures the containers of the healing agent. Some intrinsic systems, based on mechanochemistry or highly dynamic supramolecular bonds, can also exhibit autonomous behavior at room temperature [16,18,22,25].

Non-autonomous systems require an external stimulus, such as heat, light (UV), pressure, or a pH change, to activate the repair mechanism. This behavior is characteristic of most intrinsic systems, where energy input is needed to increase the mobility of the polymer chains and facilitate the reformation of dynamic bonds at the damaged interface. Although autonomy may seem more advanced, non-autonomy offers a crucial advantage: control. The user can decide when and where to initiate the repair process, which is fundamental in many applications where immediate repair is not desirable or necessary [13,18,25].

It is a common misconception to directly equate extrinsic with autonomous and intrinsic with non-autonomous. While this correlation is frequent, it is not a strict rule. The distinction is critical for application-oriented design. For example, an aerospace composite might require an autonomous system for the immediate repair of critical damage that could compromise structural integrity. Conversely, a coating for a consumer electronic device could benefit from a non-autonomous system, where scratches can be repaired “on-demand” by the user applying heat with a hot air gun [16,18,19]. Table 1 (constructed based on [13,18,25,26]) shows some paradigms of extrinsic and intrinsic self-healing processes in a comparative way according to the parameter to observe.

Occasionally, self-healing is classified as bulk or superficial. Self-healing in bulk, and for thick areas of repair, can be achieved by encapsulation, reversible chemistry, microvasculature networks, hollow fibers, and monomer phase-separation, among others. For superficial self-healing, the material must remain stable in environmental conditions such as O_2_ and H_2_O [15].

### 2.2. Performance Metrics and Healing Efficiency

To quantitatively evaluate and compare the performance of different self-healing systems, the scientific community uses a set of standardized metrics. These are crucial for determining the practical feasibility of a material for a specific application.

- Healing Efficiency: Defined as the ratio of a specific property (e.g., tensile strength, fracture toughness) of the healed material to that of the pristine material, usually expressed as a percentage. An efficiency of 100% indicates a complete recovery of the measured property [27].
- Healing Time: The duration required to achieve a certain level of property recovery under specific conditions. This parameter is critical for applications where rapid repair is required [28].
- Operating Conditions: Define the range of environmental conditions (temperature, pressure, pH, etc.) under which the healing mechanism is effective. A material that requires 120 °C to heal will not be useful in room-temperature applications [29].

In general, the type of repairing agent determines the degree of damage capable of being restored, the healing repetition capability, and the recovery rate for each technique. Healing efficiency measures the recovery of mechanical properties; healing time reflects the duration needed for repair, and operational range defines the conditions under which healing is effective.

Self-healing coatings can be defined as those that can repair a coating with no or minimal external intervention. Therefore, the self-healing approach persuades the material to be repaired (closing or sealing a defect) in situ without an external stimulus/intervention [30]. Potential areas of application of self-mending materials include coatings for corrosion protection in cars and planes (automotive and aerospace industry), aerospace nanocomposites, adhesives and sealants, regenerative medicine (artificial skin, plastic surgery), and soft robotics [31,32,33].

## 3. Self-Healing Techniques

Building on the conceptual frameworks established in the previous section, this section provides an overview of the main engineering and chemical strategies used to implement self-healing functionality in polymeric materials. These techniques are the practical manifestation of extrinsic and intrinsic principles and determine the final performance of the material. Self-healing can be achieved by encapsulation, the construction of a microvasculature network, the use of hollow fibers, encapsulation, reversible chemistry, and monomer phase-separation, among others.

### 3.1. Self-Healing by Micro and Nano Encapsulation

The pioneering work of White in 2001 suggested the use of urea–formaldehyde 200 micron microcapsules for self-healing [34]. In this work, a cyclopentadiene monomer was used to repair cracks in an epoxy matrix, which, in contact with a ruthenium-based Grubbs’ catalyst, conducted its in situ polymerization.

**Figure 3:**
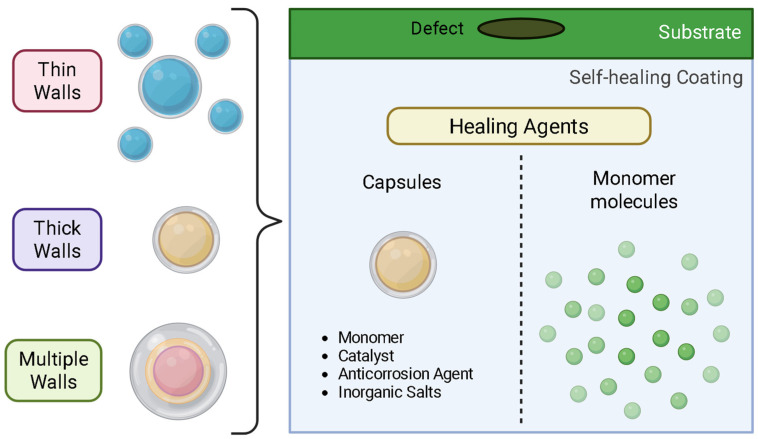
Structure of micro/nano capsules and composition of healing agents for self-healing.

Since then, autonomous self-healing uses micro/nano capsules/containers loaded with monomers or polymerizable healing agents. Capsule walls can range from simple and thin to thick, double, or multilayered structures, designed to protect sensitive chemicals and control their release for targeted or sustained action. Another class of autonomous self-healing protective coatings incorporates corrosion inhibitors, salts, monomers, and catalysts, among others, as healing agents. These are uniformly distributed in the passive matrix, keeping the healing agent embedded and avoiding premature reactions (see Figure 3). When the matrix is damaged or exposed to mechanical damage or even pH changes, the capsules respond to this signal and release encapsulated active material to heal the crack.

There are several factors to be considered when designing self-healing materials. The materials used for micro or nano encapsulation include a variety of polymers, including urea–formaldehyde, phenol–formaldehyde, polydimethyl siloxane, polyurethanes, and epoxides. Their size ranges from 30 to 200 microns for larger coatings, but for thinner coatings, nano capsules are used. The thickness of the shell/wall is also relevant, and this can be of 600–800 nm. Not only single-layer but also double-layer capsules have been suggested, especially in cases when the healing agent can be consumed even in the absence of cracks [35]. In most of these scenarios, an oil-in-water emulsion was used, where high stirring rates lead to smaller particles.

**Figure 4:**
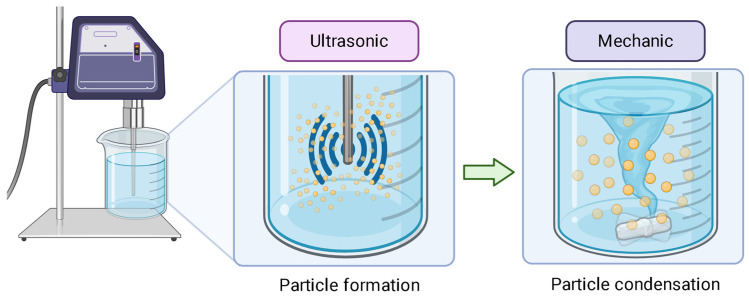
Nanocapsule formation assisted by mechanical stirring and ultrasound.

When a two-part healing system is used, an encapsulated catalyst is also needed. This can be a tin derivative used for isocyanate polymerization, or an amine for epoxides [36]. There are several methods to synthesize microcapsules, such as interfacial polymerization, coacervation, and in situ polymerization [37]. In most of these scenarios, an oil-in-water emulsion is used with high stirring rates, leading to smaller particles. The use of ultrasound also renders small particles (Figure 4). Finally, a homogeneous dispersion of the microcapsules and mechanical integrity must be achieved by controlling the agitation rate, solution viscosity, and time of dispersion. High rpm, high time for stirring, and high viscosity contribute to high capsule rupture [36].

Some of the limitations of polymers with encapsulated healing agents, while effective for rapid autonomous repair, are limited to being single-use and often fail to restore the full strength of the original material [38,39]. Furthermore, some effects of the capsules that need to be considered include their influence on mechanical properties, as a reduction in Young’s modulus and strength has been reported as the capsule concentration increases, with no clear effect of the capsule size. Adhesion strength may also vary with capsule location; for instance, poorer adhesion occurs when capsules are added to the primer, but improves when they are embedded within a middle layer of a sandwich structure. For example, Dawei reported isocyanate-containing microcapsules [40] based on a core and a double-layer shell (inner layer made of polyurea and outer layer made of polyurea formaldehyde foam).

### 3.2. Self-Healing by Polymer Inorganic Composites

Addressing the limitations of microcapsule-based strategies, polymer–inorganic self-healing composites have emerged as a mechanistically distinct platform in which functional inorganic carriers embedded within organic matrices afford stimuli-responsive, potentially multi-cycle repair [41]. In these hybrids, materials such as mesoporous silica [42], TiO_2_ [43], CeO_2_ [44], halloysite nanotubes [45], zeolites [46], and layered double hydroxides [47] are engineered to load and release inhibitors or healing agents on demand under local triggers (pH/redox gradients, ionic strength, light), thereby complementing the passive barrier provided by the binder and enabling localized restoration of functionality [41].

To situate the current state of the art and clarify relationships among material choice, healing mechanism, and performance, Table 2 compiles recent polymer–inorganic composites across substrates (e.g., Al, Cu, steels), matrices (epoxy, sol–gel, PU), and carrier chemistries. The comparison underscores how pore/lamellar architecture, interfacial compatibilization, and trigger selection govern loading capacity, release kinetics, and the persistence of the healing response.

Among the most widely employed fillers for these polymer composites are SiO_2_ nanoparticles, particularly those with mesoporous architectures (MSNs). These ceramic carriers enable inhibitor storage and stimuli-responsive release that restores interfacial functionality without relying on capsule rupture. Researchers have conducted studies to demonstrate broad applicability across substrates and pore designs [57]. On aluminum alloys (AA2024), Olivieri et al. engineered MSNs with ~2–8 nm pores to encapsulate benzotriazole (BTA), achieving pH-triggered delivery that delayed localized attack and sustained high Z values during long immersions, evidencing functional healing at defect sites [48]. On carbon steel (Q235), an epoxy coating incorporating MCM-41 nanocontainer gates release via a PEI/PSS layer-by-layer shell around a triazinyl inhibitor; the system exhibits alkaline-triggered release with three orders of magnitude gains in low-frequency impedance after 30 days, consistent with autonomous restoration at defects [58]. On mild steel, Zhu et al. introduced Zn–BTA “stoppers” on MSN pores to achieve tight, pH-responsive gating in epoxy; under acidification at active sites, the complex dissociates and releases BTA, coupling barrier recovery with inhibitor action in a controllable, on-demand fashion [59].

Conversely, halloysite nanotubes (HNTs) in polymer composites have been demonstrated to serve as pH- and stimulus-responsive nanocontainers that can be loaded with classical corrosion inhibitors and dispersed within polymer matrices to impart active, self-healing protection [45]. In recent waterborne epoxy systems, PANI-modified HNTs (BTA@PANI@HNTs) achieved ~14.5 wt.% loading and enhanced barrier/inhibition performance by 2–4 orders of magnitude in 3.5 wt.% NaCl; scratched panels exhibited minimal corrosion under salt-spray, indicating on-demand release and effective healing [60]. For carbon steel substrates, Jiang et al. reported a self-healing mechanism in imine-based epoxy thermosets reinforced with surface-modified HNTs (SiUr@HNTs); the dynamic covalent network enabled autonomous crack repair, while HNTs promoted chain mobility and stress transfer, yielding up to ~99.8% tensile strength recovery after ex situ healing, independent of inhibitor-driven anticorrosion metrics [61]. On copper, canonical demonstrations of MBT/MBI/BTA@HNTs in polyurethane/acrylic coatings established efficient inhibitor delivery at defect sites and durable scratch-healing behavior, a benchmark now widely referenced [62].

Zeolitic- and TiO_2_-based inorganic polymeric composites have likewise enabled extrinsic, triggerable self-healing in coatings by coupling high storage capacity with selective release at defect sites. Building on this paradigm, zeolites have been deployed as dual-nanocontainer architectures that produce pH-responsive release profiles and sustain impedance on aluminum, illustrating how pairing a zeolite reservoir with a biopolymer gate can prolong the self-healing window [50]. Broader surveys of zeolite pigments further underscore their role as environmentally safer carriers whose ion-exchange kinetics and framework chemistry can be tuned to synchronize release with corrosion-induced pH/ionic shifts [56].

For TiO_2_, nanotubular and core–shell morphologies have been leveraged both as inhibitor hosts and as reactive reservoirs for monomers/curing agents that polymerize in situ to close cracks. A representative strategy combines TiO_2_ nanotubes (epoxy pre-polymer) with mesoporous silica (amine hardener) to achieve chemical “healing” of scratches on carbon steel via delayed co-release and cross-linking within the defect [63]. More recent TiO_2_-based capsules loaded with benzotriazole, as well as related inhibitors integrated into epoxies, exhibit multi-cycle, stimulus-responsive recovery of electrochemical performance on steels and Al alloys, aligning release/gating chemistry with measurable restoration at damage sites [64].

Within polymer–inorganic self-healing architectures, epoxy resins remain the workhorse matrix owing to their ability to disperse nanocontainers efficiently, preserve adhesion to metallic substrates, and accommodate multifunctional additives that enable on-demand repair. Recent designs demonstrate that tailoring carrier composition and gating chemistries within epoxy governs loading capacity, leakage suppression, and trigger ability [65].

Effective deployment of the nanoparticle strategies outlined above requires an appropriate polymer matrix that (i) is compatible with and disperses inorganic carriers, (ii) transmits damage-borne triggers to those carriers, and (iii) permits transport and retention of released species at defect sites. Comparative investigations consistently identify epoxy as the predominant platform for smart coatings due to its strong metal adhesion, chemical/thermal stability, and tolerance for high micro/nanocontainer loadings without catastrophic loss of film integrity; critically, matrix chemistry and crosslink density modulate both carrier dispersion and the efficiency of stimulus–response processes [1].

In parallel, hybrid epoxy–sol–gel (ORMOSIL/Zr-based) networks are increasingly employed to tailor interfacial bonding and permeability, thereby enhancing retention and on-demand release in active systems [66]. Broad, cross-disciplinary surveys of corrosion-responsive self-healing coatings likewise indicate that most validated case studies (spanning MSN, LDH, zeolite, and HNT carriers) are implemented in epoxy or epoxy hybrid binders, reinforcing epoxy’s status as the “workhorse” matrix for reproducible, multi-cycle autonomous repair [33].

A critical assessment of recent advances also reveals non-trivial limitations that hinder the translation of inorganic polymeric self-healing coatings to service environments. Notably, the carrier attributes that enable controlled release (high surface area and open porosity) can also induce premature leaching under fluctuating humidity, salinity, or pH, thereby shortening the effective healing window [67].

Similarly, investigations into stimuli-responsive coatings underscore that although nanocontainers enhance barrier recovery after localized failure, both loading capacity and trigger specificity are inherently limited; mismatched triggers (e.g., background pH drift) can induce sub-optimal dosing or false positives, ultimately degrading multi-cycle performance [68].

A second set of constraints arises from formulation and processing. Achieving uniform dispersion of rigid inorganic carriers at practical volume fractions without agglomeration is challenging; insufficient interfacial compatibilization increases viscosity, seeds micro voids, and may compromise adhesion and toughness of the host matrix. These effects have been documented for epoxy nanocomposites and highlighted in recent synthesis–structure–property studies of self-healing systems [69]. Even when initial dispersion is satisfactory, re-agglomeration during cure or service can localize stresses and reduce the elastic modulus, with measurable penalties in thermomechanical response reported for nanoparticle-filled polymers [70]. Such processing sensitivities complicate scale-up, where shear histories and solvent-removal profiles diverge from laboratory conditions, and where multilayer architectures must balance permeability (to deliver triggers and species) against barrier integrity.

Polymer–inorganic self-healing composites integrate the passive barrier function of polymer binders with the active, triggerable behavior of inorganic carriers (mesoporous silica, HNTs, zeolites, LDHs, TiO_2_), enabling localized functional restoration via on-demand release and reversible interfacial chemistries. Across aluminum, copper, and steel substrates, recent studies show that pore/lamellar architecture, interfacial compatibilization, and matrix selection, particularly epoxy and epoxy–sol–gel hybrids, govern loading capacity, leakage suppression, release kinetics, and cycling durability. Nevertheless, key gaps remain, including the establishment of standardized healing metrics, long-term agent retention without toxicity, scalable dispersion and processing routes, and rigorous validation under coupled electrochemical, mechanical, and environmental stressors.

### 3.3. Self-Healing by Reversible Chemistry

In reversible-chemistry systems, self-healing can be achieved without embedded carriers: recovery arises from dynamic covalent (or supramolecular) bonds that break and reform under external stimuli such as heat, light, pH, or catalysts, enabling local network rearrangement that closes cracks and restores continuity [71]. Two major classes of these materials have been studied: covalent adaptable networks (CANs) and vitrimers. CANs encompass both dissociative exchanges (bond rupture followed by re-formation, typically lowering crosslink density during activation) and associative exchanges (bond-exchange with preserved crosslink density), enabling malleability, welding, and repeated healing in crosslinked polymers [72]. Vitrimers represent the associative limit, displaying Arrhenius viscosity and a topology-freezing temperature (Tv) that demarcates solid-like service from malleable reprocessing regimes [73].

In coatings, these intrinsic mechanisms contrast with Section 3.2, polymer–inorganic composites by eliminating mobile reservoirs, instead programming reversibility into the matrix itself, thereby reducing leaching risks while demanding careful control of exchange chemistry, activation temperature, and network architecture. Recent investigations and demonstrations detail these paradigms and their relevance to protective films and adhesives, including advances in epoxy vitrimers, low-Tv designs, and sustainability-oriented dynamic networks [16,74].

Covalent adaptable networks (CANs) provide an intrinsic self-healing route in which the cross-links of the polymer are dynamic, obviating the mobile reservoirs required by polymer composites and instead programming bond exchange directly into the matrix. Extensive studies have established CANs based on reversible Diels–Alder adducts and other exchange chemistries that impart malleability, weldability, and damage repair to otherwise permanent networks, and subsequently formalized the vitrimer concept as the associative limit of CANs [75].

**Figure 5:**
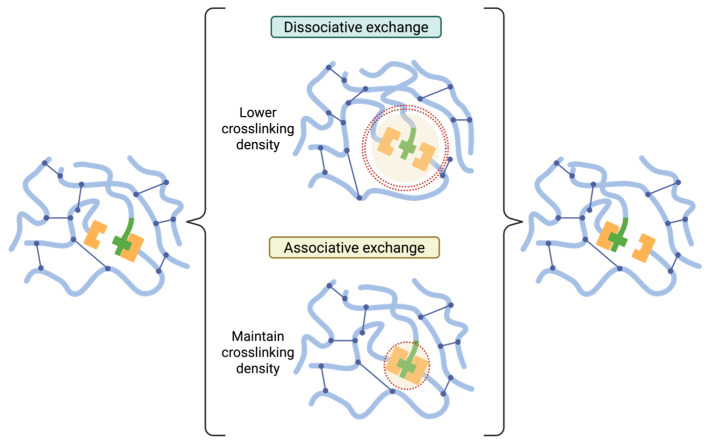
Schematic of reversible chemistry in CANs: dissociative exchange and associative exchange.

The general reaction mechanisms for CANs are depicted in Figure 5. In dissociative exchange, covalent bonds are cleaved to regenerate latent functional groups that later recombine at new positions; during activation, the transient reduction in cross-link density can diminish dimensional stability and solvent resistance. In associative exchange bond scission occurs concertedly with bond formation, preserving cross-link density while allowing network topology to relax; cracks can therefore close and interfaces re-form without global softening [76]. At the coating scale, both pathways facilitate stress relaxation at the damage tip, interfacial re-stitching, and recovery of barrier/adhesive functions once the exchange reaction is activated by heat, light, catalysts, or pH.

The variety of materials leveraging intrinsic self-recovery is broad, encompassing both associative and dissociative exchange mechanisms. As a prototypical associative case, boronic–ester exchange in silicone networks affords autonomous scratch repair and outstanding durability even at sub-10 nm thicknesses, providing direct evidence that bond exchange alone can reconstitute surface functionality in coating-relevant films [77]. Extending to structural applications, epoxy vitrimers that rely on transesterification (or synergistic exchanges such as disulfide metathesis coupled with carboxylate transesterification deliver rapid and repeatable strength recovery alongside reprocessability; notably, complete healing within minutes and excellent mechanical retention are achieved in low-Tv designs that activate at modest temperatures compatible with coated substrates [78].

Recent studies translate the same principles to carbon-fiber/epoxy laminates, where thermal activation of dynamic bonds closes microcracks and scratches, extending service life as verified by microscopy and mechanical cycling [79]. Complementarily, bio-based epoxy systems that integrate imine and disulfide exchanges enable solvent-free healing and recyclability, underscoring the feasibility of sustainability-oriented dynamic networks for protective films and adhesives [80].

Vitrimers offer repeated damage repair, restoration of molecular-level integrity, and retention of high mechanical strength while resisting environmental degradation. Their primary limitation is the relatively high activation temperature; however, this can be reduced through appropriate catalysts, thermally conductive nanostructures such as graphene, or multi-stimuli responsive approaches (e.g., light, pH) [81].

A wide range of polymers has been adapted to vitrimer chemistry, enabling application-specific coatings and films. Representative systems include: epoxy vitrimers, classically obtained via transesterification of epoxy–acid networks, which exhibit malleability, solvent resistance, and repeatable strength/adhesion recovery with closed-loop recyclability [82]; PDMS vitrimers based on boronic ester exchange, which yield ultrathin, durable, scratch-healing coatings with sustained surface functionality [83]; polyurethane vitrimers employing transcarbamoylation or complementary dynamic bonds, achieving strong self-healing and closed-loop recyclability in film geometries [84]; and polyimine vitrimers (Schiff-base exchange), which deliver room-to-moderate-temperature healing, welding, and reprocessability, including bio-derived formulations relevant to protective layers and adhesives [85]. Collectively, these platforms illustrate how judicious selection of the base polymer and exchange chemistry enables vitrimer coatings to balance activation temperature, mechanical retention, and healing rate without the inherent leaching risks of carrier-based systems.

#### 3.3.1. Epoxy-Based Vitrimers

Epoxy-based vitrimers display outstanding self-healing capabilities, autonomously repairing microcracks and restoring mechanical properties under appropriate stimuli such as heat or light. In these systems, transesterification, disulfide exchange, imine exchange, or thiol-ene click reactions allow the network to perform as a conventional thermoset in service while enabling repair at elevated temperatures or above the glass-transition temperature. In addition, incorporating bio-derived feedstocks such as lignin or rosin mitigates environmental concerns associated with conventional epoxy resins [86].

In re-processable epoxy vitrimer adhesives, zinc-carboxylate-catalyzed transesterification supports repeated bond/de-bond cycles and strength recovery with minimal performance loss, demonstrating practical welding/repair workflows compatible with coating maintenance [87]. Kinetic analyses of epoxide–thiol/ester architectures link molecular exchange rate constants to macroscopic flow and stress relaxation, offering actionable guidance to set Tv and healing times that align with metal substrates and paint-oven conditions [88]. To further lower activation temperatures, epoxy networks that integrate auxiliary dynamic motifs (disulfide metathesis and hydrogen bonding) achieve autonomous or near-ambient healing while maintaining solvent resistance, a valuable combination for temperature-sensitive substrates and field repairs [89].

From a sustainability standpoint, bio-derived epoxy vitrimers and vitrimer composites enable closed-loop recycling, reprocessability, and solvent-free repair, thereby aligning intrinsic self-healing with circularity targets in protective films [90]. Finally, in fiber-reinforced epoxy systems, vitrimer exchange reactions restore matrix continuity at microcracks and interfaces, extending fatigue life and enabling thermal welding or reshaping of cured laminates, capabilities validated by microscopy, mechanical cycling, and post-repair property retention [91]. In conclusion, recent studies on epoxy-based vitrimer coatings have demonstrated that dynamic covalent networks can deliver repeatable self-healing, recyclability, and even bio-based formulations while maintaining barrier and mechanical performance [92]. Nevertheless, most of these systems still require relatively high activation temperatures or external intervention to trigger bond exchange, and increasing network mobility often comes at the expense of stiffness and long-term creep or fatigue resistance [86]. In addition, the durability of vitrimer coatings under realistic service conditions (cyclic humidity, UV radiation, aggressive chemicals) and their processability at industrial scale remain open issues [93].

Therefore, current research is increasingly focused on catalyst- and solvent-free, bio-based epoxy vitrimers with lower activation energies, multi-stimuli responsiveness, and optimized network architectures that balance mechanical robustness, healing efficiency, and environmental sustainability.

#### 3.3.2. PDMS Vitrimers

Polydimethylsiloxane vitrimers embed associative bond exchange directly into silicone networks, enabling crack closure via network topology rearrangement while preserving instantaneous crosslink density, hydrophobicity, and optical clarity. Their viscosity follows Arrhenius behavior, with a programmable topology-freezing temperature (Tv) that delineates the healing/reprocessing window without compromising dimensional integrity; consequently, coating-relevant films can undergo repeated repair without the leaching risks associated with extrinsic carriers [94].

**Figure 6:**
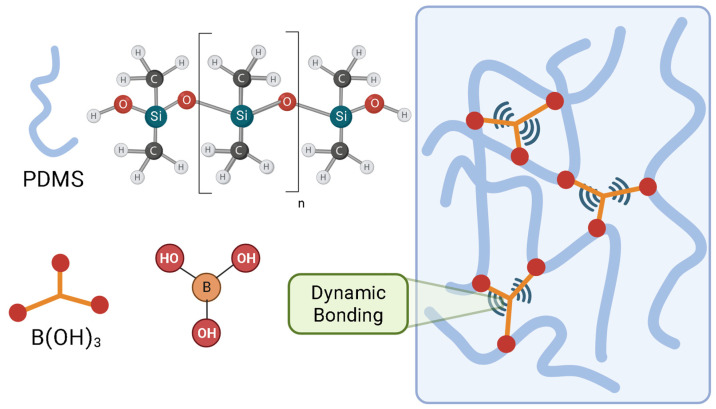
Schematic of dynamic boronic ester exchange in PDMS vitrimer coatings.

In PDMS vitrimers, stress concentration at a scratch activates local bond exchange, allowing strands to migrate and re-form crosslinks across the gap, thereby restoring continuity. Figure 6 illustrates this associative mechanism, which maintains crosslink density while enabling stress relaxation and interfacial re-stitching under mild thermal activation [95].

Among PDMS-based vitrimers, there has been particular interest in ultra-thin films (<10 nm) cross-linked via boronic ester chemistry. These coatings exhibit autonomous surface healing under gentle heating and retain hydrophobicity after repeated scratching, cutting, and condensation cycles, direct evidence of carrier-free self-repair in transparent coatings [77]. Progressing toward thicker layers and added functionality, elastic boronic ester vitrimers offer tunable mechanics and recyclability through systematic variation in boronic substituents, providing a design handle to align healing rate and Tv with service conditions [96].

In parallel, silyl ether vitrimer chemistry offers an alternative for applications demanding higher thermal stability, enabling reprocessable, self-healing silicone networks via direct silyl ether metathesis [94]. PDMS vitrimer layers have also been integrated into eco-efficient, self-stratifying coating stacks employing bio-based binders, yielding hydrophobic films that recover from scratches while remaining compatible with scalable processing [97].

Collectively, PDMS-based vitrimers translate associative exchange into coatings that pair carrier-free self-repair with the hallmark attributes of silicones—low Tg, flexibility, hydrophobicity, and optical clarity—while allowing precise tuning of the activation window through functional group selection, catalyst loading, and the topology-freezing temperature. In practice, this enables transparent, ultrathin films that repeatedly recover from surface defects under mild heating, as well as elastomeric overcoats that weld, reshape, and preserve barrier/wetting performance after damage. Taken together, these investigations position PDMS vitrimers as versatile, scalable binders for reliable, multi-cycle self-healing coatings under demanding service conditions, complementing the epoxy vitrimer platforms discussed above.

Overall, recent studies on PDMS-based vitrimer coatings and elastomeric layers have demonstrated that dynamic silicone networks can deliver ultra-thin, fluorine-free hydrophobic films with autonomous damage recovery and re-processable, malleable elastomeric phases for flexible devices [98,99]. Nevertheless, most PDMS vitrimers still rely on thermally activated bond exchange and must balance high chain mobility for rapid self-healing with sufficient crosslink density to avoid excessive creep and loss of mechanical integrity over time [100].

#### 3.3.3. Polyurethane Vitrimers

Leveraging reversible dynamic covalent bonds, polyurethane vitrimers exhibit efficient self-healing and repair across diverse conditions—including ambient and elevated temperatures, thermo-compression, UV irradiation, microwave exposure, saline environments, and alkaline/acidic media—while maintaining functional integrity [101]. In several studies, polyurethane vitrimers have shown their self-healing performance across chemistries and formats: renewable castor-oil–based PU vitrimers that heal, self-weld, and are reprocessable via catalyst-mediated transcarbamoylation (thermally induced dual-shape memory and self-welding) [102]. Waterborne polyurethane–urea coatings incorporating dynamic hindered urea bonds achieve robust mechanical properties and intrinsic self-healing, even under saline conditions, highlighting applicability to protective films [103,104].

Green chemistry has also extended to PU materials. Non-isocyanate PU vitrimers based on cyclic carbonates combine degradability, recyclability, and efficient self-repair, offering a more sustainable route to vitrimeric coatings [104]. Green chemistry has also extended to PU materials. Non-isocyanate PU vitrimers based on cyclic carbonates combine degradability, recyclability, and efficient self-repair, offering a more sustainable route to vitrimeric coatings. Polyhydroxyl urethanes vitrimers can be prepared from five- or six-membered cyclic carbonates that react with multifunctional amines without catalyst, surfactants, and stabilizers [105]. Hybrid designs further broaden functionality; for example, organic–inorganic PU vitrimers reinforced with UPy-modified SiO_2_ nanofillers couple vitrimer exchange with supramolecular interactions to enhance strength, solvent resistance, and autonomous healing [106].

In parallel, formulations derived from phenolic carbamate or rosin lower activation temperatures and advance bio-based circularity while preserving reprocessability and reliable self-repair [107]. Collectively, these studies position PU vitrimers as adaptable binders for protective coatings that demand multi-cycle healing under diverse service conditions. In Table 3, the different polyurethane types of bond exchanges in vitrimers are presented.

Similarly, catalyst-free or self-blown foamed polyurethane vitrimers can be prepared by the blocking reaction of dioximes or trioximes with isocyanates or by decarboxylation of oxamic/polyoxamic acids [113,114].

In summary, recent studies on polyurethane vitrimer networks and coatings have shown that dynamic urethane, carbonate, and related exchangeable linkages can provide self-healing, recyclability, or re-processability, and even bio-based or non-isocyanate formulations while retaining satisfactory mechanical and barrier performance [115,116]. However, many systems still require relatively high activation temperatures, suffer from creep when network mobility is increased, and lack long-term validation under realistic service conditions or scalable implementation on complex geometries [109].

#### 3.3.4. Polyimine Vitrimers

Polyimine vitrimers employ dynamic imine exchange as the driving mechanism for intrinsic, carrier-free self-healing, welding, and reprocessability. In contrast to inorganic composite systems (Section 3.2), where repair depends on the mobility of an encapsulated agent, polyimine networks encode associative transamination directly into the backbone, allowing bond exchange under mild heating, or even near ambient conditions, to promote stress relaxation and crack closure without reducing instantaneous cross-link density. Notably, this chemistry is also chemically circular: the same C=N linkages that rearrange topologically can be selectively cleaved or solvated to enable closed-loop recycling [117]. These advantages have accelerated the development of this family for thin films [118], adhesives [119], and coating-relevant matrices [120]. In parallel, multiple studies have clarified how cross-link density, imine functionality, and network architecture govern relaxation times, activation windows, and mechanical retention [121].

Recent demonstrations further highlight coating-relevant performance and design. Catalyst-free, bio-based polyimine vitrimers derived from renewable aldehydes exhibit rapid healing, malleability, and solvent-assisted recyclability while retaining thermal stability [122]. Complementarily, epoxy–amine polyimine networks synthesized from vanillin-based diimine–diglycidyl monomers offer an aromatic pathway to vitrimeric behavior in thin films and adhesive formats, with relaxation times and topology-freezing windows tunable via network architecture and stoichiometry [110]. At the same time, chitosan/vanillin polyimine vitrimers show that fully or largely bio-derived formulations can deliver multi-stimuli responsiveness (self-healing, weldability) under mild conditions, advancing greener binder chemistries for protective coatings [123].

Together, polyimine vitrimers offer a compelling suite of attributes for coatings: carrier-free self-repair via associative transamination, rapid welding at mild temperatures, chemical recyclability of the C=N network, and broad access to bio-based feedstocks, all while maintaining sufficient thermal and mechanical robustness for thin films and adhesive interphases.

Across CANs and vitrimers, reversible-chemistry coatings replace mobile reservoirs with programmed bond exchange within the matrix, delivering repeatable crack closure, welding, and reprocessing while preserving cross-link density in the associative limit (vitrimers) and enabling lower-temperature activation in selected dissociative systems [82]. Their key limitations can be framed as follows: (i) activation windows, requiring alignment of healing rate with service stability and creep suppression; (ii) chemistry sensitivities; and (iii) comparability and scale-up, namely the need for standardized metrics of healing efficiency under corrosive, thermal, and mechanical cycling, together with processing routes that preserve dispersion, adhesion, and surface quality in thin films [124]. With these constraints explicitly addressed, reversible-chemistry coatings are well positioned to complement polymer–inorganic composites, enabling reliable, multi-cycle self-repair in protective films and adhesive layers across demanding service conditions.

Overall, polyimine vitrimers represent a versatile family of dynamic covalent networks in which imine metathesis enables reshaping, welding, and self-healing while preserving the dimensional stability of conventional thermosets [121]. Recent follow-up studies on polyimine vitrimer and composites have shown that dynamic imine exchange can impart robust mechanical strength, rapid self-healing, re-processability, closed-loop recyclability, and often fully bio-based formulations [125]. However, many imine vitrimers still require relatively high temperatures or long heating cycles to activate bond exchange, and the intrinsic susceptibility of C=N bonds to hydrolysis raises concerns about long-term stability in humid or aqueous environments [126]. Current efforts, therefore, focus on more hydrophobic or aromatic imine architectures, multi-dynamic networks, and catalyst-free, bio-based chemistries that lower activation energies while improving water resistance and enabling durable coating applications [127].

In comparison, epoxy vitrimers generally offer high mechanical robustness and excellent adhesion, making them ideal for protective coatings, but they often require elevated temperatures for activation. PDMS-based vitrimers show superior flexibility, hydrophobicity, and optical transparency, enabling efficient surface scratch repair, though they exhibit lower modulus. Polyurethane vitrimers combine mechanical toughness with versatile chemistries (transcarbamoylation, hindered urea, imine exchange), supporting ambient-to-moderate healing and high durability. Polyimine vitrimers offer rapid healing and chemical recyclability but may show sensitivity to moisture or hydrolysis depending on formulation. This comparison clarifies how the choice of dynamic bond chemistry governs the balance between healing rate, activation temperature, environmental resistance, and application-specific performance [76,80,119,122,128,129].

### 3.4. Self-Healing by Microvasculature Formation

**Figure 7:**
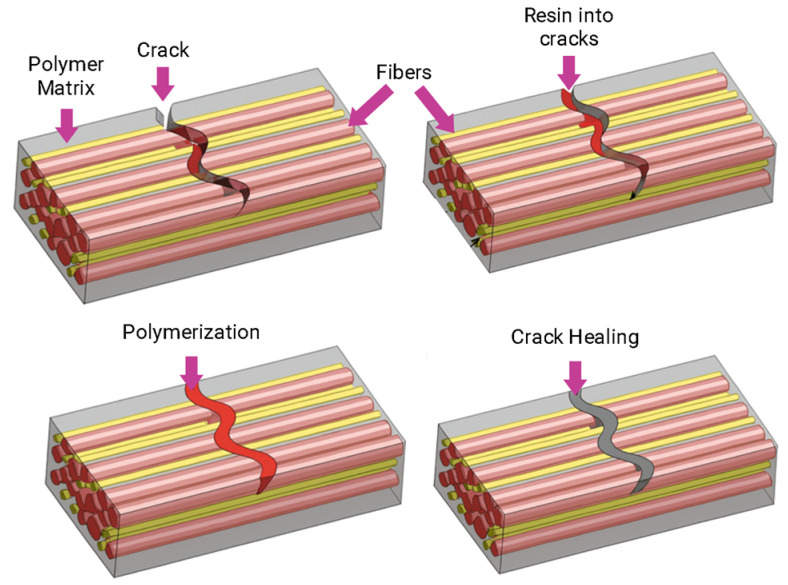
Representation of a self-healing by hollow fibers in a fiber-filled polymeric matrix.

Microvascular materials are based on microscale channels embedded in polymer substrates. These types of materials have gained significant attention in recent years, since they can effectively extend the service life of materials when applied as coatings. Microvascular systems can autonomously repair damage multiple times by delivering healing agents through channels to crack sites. Some classical approaches, which are based on microcapsules or hollow fibers, only provide a single healing cycle, with limited control over agent delivery; in contrast, microvascular systems address these limitations by embedding interconnected channels within the coating, enabling multiple repair cycles and targeted healing agent transport (Figure 7) [130].

#### 3.4.1. Microvasculature Architecture

Polymer-microvasculature interactions are governed by microarchitecture and physicochemical properties. These features need to be tailored for a given application, such as protecting coatings in automotive, drug-delivery systems, and tissue engineering applications. Therefore, microvascular networks vary in complexity, from simple single-line channels to fully interpenetrating three-dimensional designs. For example, Gariboldi et al. (2019) demonstrated that bio-ceramic scaffold architecture at the hundreds-of-microns scale significantly influences microcapillary-like structure formation, with grooves of 330–600 μm width and 150–585 μm depth producing highly aligned structures up to 5 mm long [131]. Furthermore, Toohey et al. (2007) developed a bio-inspired self-healing system using three-dimensional microvascular networks embedded in substrates to deliver healing agents to polymer coating cracks, enabling repeated autonomous repair [132]. More examples can be found in Table 4, where a summary of key studies on microvascularized coatings for surface protection is shown. The type of system employed, the materials used, relevant experimental details, and targeted application are highlighted.

As observed, key design parameters include channel diameter; other, less specified, parameters are density and connectivity. Altogether, these aspects govern healing efficiency and mechanical impact. Despite the substantial progress in the production of a variety of architectures in different applications, there is no universally accepted framework for the characterization and reporting of microvascular self-healing coatings. Standard guidelines would enable more consistent data interpretation and facilitate analysis across studies. Additionally, the integration of theoretical simulations, artificial intelligence (AI), and machine learning methodologies can accelerate the development of classification systems and optimize the design of self-healing composites by identifying correlations between structural topology and healing performance [139].

#### 3.4.2. Techniques to Produce Microvasculature Networks

Microvasculature fabrication remains a critical challenge in tissue engineering, requiring innovative techniques to create functional vascular networks at a cost. Several engineering approaches have emerged to address this need. For example, thermal inkjet printing technology enables precise fabrication of micro-sized fibrin channels, where human microvascular endothelial cells (HMVECs) align and proliferate to form confluent linings with 3D tubular structures [140]. Another method is the direct-write assembly of colloidal ink, which is extruded through a micronozzle to create the desired 3D network architecture [141]. The polymer is then infused around the wax, which is later removed by melting. Another popular method is the sacrificial fibers method [142], in which a thin polymer filament is embedded within a composite material during manufacturing. After the material cures, the fibers are removed by heating or dissolving to create hollow channels.

Furthermore, 3D printing/additive manufacturing has rapidly gained relevance in recent years due to its exceptional versatility and design freedom [143]. Advanced additive manufacturing (AM) techniques enable the precise printing of complex bio-inspired vascular networks. For instance, AM techniques have been applied to the fabrication of biomimetic vascular networks for tissue regeneration. In aerospace and structural applications, AM enables the creation of embedded microvascular cooling or self-healing networks that enhance thermal regulation and extend the operational life of lightweight composite structures [144,145]. In the energy sector, different sorts of microvascular architectures produced via AM have been integrated into heat exchangers and electrochemical devices, promoting efficient heat transfer and fluid distribution within compact geometries. Similarly, in the energy field, conductive vascular architecture produced with AM has been implemented in the construction of solar cells and batteries [146]. Overall, these advancements in AM show that microvascular design, once devoted to self-healing polymers, now serves as a broad engineering framework for enhancing the functionality and resilience of next-generation coating systems.

Electrospinning is another recurring technique used for fabricating microvascular self-healing coatings, enabling fine control over fiber morphology, porosity, and the spatial distribution of healing agents. Specifically, through coaxial electrospinning, where core–shell nanofibers can be produced, and the inner core encapsulates liquid healing agents. For example, electrospinning has been used for vascular restoration through the fabrication of electrospun dimpled hollow fibers as a scaffold for HUVEC cells [147]. Another example is the work of Vintila et al. (2022), who demonstrated that coaxial electrospinning can be used to fabricate core–shell (dicyclopentadiene/polyacrylonitrile) nanofibers enabling controlled release and autonomous crack repair within polymeric coatings that mimic natural microvascular systems [133].

#### 3.4.3. Performance Testing

The development of self-healing materials has largely focused on demonstrating the ability to repair damage under controlled, single-event conditions. While these initial studies are essential to validate the concept, they provide limited information about the material’s performance under realistic service conditions. In practical applications, structures and coatings are often subjected to repeated mechanical stress, environmental variations, and cumulative damage over time. Therefore, it is critical to evaluate both the endurance of the healing system, its ability to recover repeatedly, and its performance, including the efficiency, speed, and completeness of healing. Measuring endurance and performance through systematic testing allows researchers to identify limitations such as the depletion of healing agents, degradation of microvascular networks, or reduced mechanical recovery after multiple cycles.

A comprehensive evaluation of microvascular self-healing coatings requires assessment beyond single-event repair. Key performance aspects include the following: (i) multi-cycle healing efficiency, quantifying the ability of a system to sustain successive healing events without depletion of the healing agent; (ii) healing kinetics, measured through fracture recovery, impedance restoration, or barrier recovery time; (iii) environmental ageing, including UV exposure, salt spray, humidity cycling, and thermal cycling; and (iv) mechanical fatigue, where coatings undergo repeated loading to determine the persistence of vascular integrity. Recent studies demonstrate that the architecture of the vascular network strongly influences durability. For example, one PDMS-based self-healing polymer system achieved over 90% toughness recovery after multi-cycle healing. Another study of dual dynamic covalent networks demonstrated high residual strength following accelerated degradation [148,149,150]. These evaluations highlight the need for more strict standardized protocols to compare systems on equal terms, as current literature uses widely varying defect geometries, flow rates, and environmental conditions [27,38,39,67,121,151,152,153,154,155].

In Table 5, a survey of various microvascular self-healing coating systems is shown, highlighting their architecture, substrate or capsule materials, reported life cycles, and application. In this sense, it is important to notice how relevant studies report the extended performance and endurance of these coatings.

It can be noticed that there is a need to accurately evaluate the long-term performance and reliability of self-healing systems. Furthermore, materials should be tested under conditions that mimic real environments: exposure to temperature changes, moisture, UV, mechanical fatigue, or chemical corrosion, as appropriate. Such ageing or environmental degradation can reduce the availability or reactivity of healing agents or damage vascular networks. Validating healing kinetics (how fast healing occurs), durability, and stability of the healing system over time is, therefore, a major opportunity in the development of self-healing materials.

## 4. Self-Healing Applications

### 4.1. Self-Healing for Corrosion Protection

**Figure 8:**
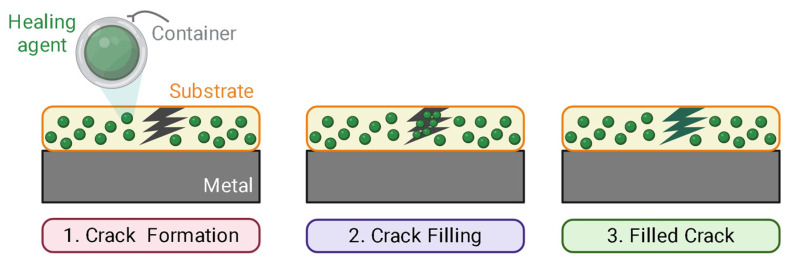
Schematic of extrinsic self-healing in inhibitor-loaded container coatings on metal substrates.

Corrosion imposes a persistent, multi-trillion-dollar burden on infrastructure and manufacturing; the widely cited NACE IMPACT study estimated a global annual cost near USD 2.5 trillion, underscoring both the scale of the problem and the value of proactive corrosion management [161]. Conventional organic coatings function primarily as passive barriers, and once they are breached by scratches, pinholes, or underfilm delamination, corrosion localizes and accelerates at the defect. By contrast, self-healing anticorrosion coatings are engineered to sense damage and autonomously restore protection through inhibitor release or dynamic bond exchange, shifting the paradigm from static to damage-responsive protection [33]. Recent investigations emphasize that such “active” coatings can maintain high impedance and suppress anodic/cathodic processes over multiple damage cycles when their healing chemistries are matched to the service environment [162]. A schematic sequence of the extrinsic micro/nanocontainer approach is provided in Figure 8.

Two complementary strategies currently dominate self-healing anticorrosion design. In extrinsic systems, micro/nanocontainers store and release corrosion inhibitors or polymerizable agents in response to stimuli (pH, redox, chloride, and light), thereby re-passivating or rebonding the damaged region [71]. Principal carrier materials used to tailor these functions include mesoporous silica [163], halloysite nanotubes [62], zeolites [164], layered double hydroxides [165], and TiO_2_ [166].

Inhibitors can also be mixed directly into the polymer matrix—for example, lithium carbonate in polyurethane coatings [167]. However, to avoid salt aggregation and undesirable side reactions, composite polymer coatings are often preferred. Several approaches have been developed depending on the substrate (stainless steel, aluminum, and magnesium) and the base coating composition. As a representative case, Cho et al. [168] used droplets of a siloxane monomer while encapsulating dibutyltin dilaurate; when the monomer tends to react with the catalyst, both can be co-encapsulated. The PDMS-based healing agent comprises 96 vol% hydroxyl end-functionalized polydimethylsiloxane (HOPDMS) and 4 vol% polydiethoxysiloxane (PDES), whereas the catalyst solution contains 5 wt.% DMDNT in chlorobenzene. To avoid premature reaction with amines in the epoxy matrix, urea–formaldehyde microcapsules were employed.

Conversely, intrinsic systems embed reversible chemistry directly into the binder (vitrimeric epoxies or polyurethane/imine networks), enabling crack closure and barrier recovery without mobile payloads; although these matrices do not dispense inhibitors, they can restore film continuity and, when paired with primers or inhibitor packages, stabilize the interface after damage [169].

Table 6 summarizes the state of the art for representative self-healing materials, both extrinsic and intrinsic, highlighting the most relevant features and performance metrics reported in each study.

Across aluminum and steels, mesoporous silica (MSNs) remains a versatile platform because their 2–10 nm porosity and surface chemistry allow both high inhibitor loading and triggered dosing at defects. On AA2024, Olivieri et al. embedded MSNs (≈2–8 nm) loaded with benzotriazole (BTA) into polymer films and showed pH-responsive release that sustained high |Z| over prolonged immersion, delaying localized attack, clear evidence of functional healing in an aerospace-relevant alloy [48]. Complementing this, Zhu et al. engineered Zn–BTA “stoppers” on MSN pores so that BTA is tightly retained but liberated under acidification at active pits; in epoxy on mild steel, this design delivered controllable, on-demand recovery of barrier properties and reproducible EIS gains [59]. PEI-wrapped MSNs have likewise been used to sharpen pH sensitivity and suppress premature leakage in steel-focused coatings [49]. Beyond classical BTA reservoirs, hybrid MOF@SiO_2_ containers, like BTA-ZIF-8@SiO_2_ in sol–gel/epoxy hybrids on Al, combine a MOF core with a silica shell, achieving multi-cycle EIS recovery and scratch healing [170].

Transferring this concept to waterborne systems, PANI-modified HNTs incorporated into epoxy for steel panels reached ~14.5 wt % BTA payload and delivered 2–4 orders-of-magnitude increases in |Z| in 3.5% NaCl; crucially, scribed specimens exhibited minimal underfilm attack in salt spray, confirming pH-triggered healing in an industrially relevant binder [60]. In parallel, epoxy–ester coatings containing HNT@imidazole–dicarboxylate on carbon steel corroborated these trends with higher low-frequency impedance and slower rust propagation, showing that the lumen-release paradigm can be adapted across chemistries without sacrificing barrier integrity [172].

While HNTs and MSNs rely on encapsulation and release, layered hosts like LDH/LHS enable anion-exchange-driven self-healing that is naturally coupled to chloride ingress. A zinc-layered hydroxide salt intercalated with molybdate (MoO_4_^2−^) in epoxy on carbon steel provided active, defect-selective protection, with self-healing verified by EIS/SVET and suppression of pit growth, thereby validating LDH-type hosts as “smart reservoirs” that respond to corrosive flux rather than passive time [173]. Extending this platform, LDH films intercalated with vanadate, phosphate, or amino-acid-derived anions have been tuned for release kinetics and film-forming behavior, broadening applicability from steels to light alloys and highlighting how interlayer chemistry can be used to synchronize dosing with the corrosion micro-environment [174].

Crystalline microporous zeolites offer a third, complementary route in which ion-exchange capacity is paired with polymeric “gates” to refine trigger fidelity. On aluminum, an epoxy using ZSM-5 + chitosan realized pH-responsive release with sustained impedance and an extended healing window, evidencing that combining a robust inorganic reservoir with a bio-gate stabilizes delivery under fluctuating wet/dry cycles [50]. In a related vein, NaY-type zeolites co-loaded with Ce^3+^ and DEDTC in epoxies have blended cathodic passivation with anionic inhibition on AA2024-T3, illustrating that mixed-mode actions can be engineered within one pigment to address both anodic and cathodic reactions at defects [171]. Together with broader surveys identifying zeolite pigments as eco-safer substitutes for chromates, these examples reinforce the role of framework chemistry and ion exchange in achieving durable, active coatings [56].

At incipient defects, corrosion rapidly shifts local pH and potential away from bulk conditions; self-healing coatings harness these gradients to trigger inhibitor dosing or bond-exchange reactions, thereby restoring passivity and sealing transport pathways. Building on the materials and mechanisms discussed above, application-oriented research now spans multiple sectors, including marine transportation [175], electrical insulation [176], and aerospace [177]. In Table 7, anticorrosive self-healing materials across different industrial sectors (automotive, marine, aerospace, energy/civil infrastructure, and electronics) are presented.

Despite significant progress, several intertwined constraints still govern the actual application of these anticorrosive self-healing materials. First, payload management is delicate: the same high surface area and open porosity that enable large inhibitor loadings can also promote premature leaching under fluctuating humidity, temperature, and salinity, narrowing the effective healing window; careful choice of gates (metal–inhibitor complexes, polyelectrolyte shells) mitigates this but may slow response or complicate processing [164]. Second is the trigger specificity matters. Background pH drift, passive oxide dissolution, or galvanic coupling can cause misdosing, diminishing multi-cycle efficacy; dual-container architectures improve robustness but add interfaces and cost [33].

Dispersion and scale-up represent one of the principal challenges for these materials, keeping rigid inorganic carriers uniformly dispersed at practical volume fractions without raising viscosity, seeding micro voids, or degrading adhesion requires compatibilizers and well-controlled cure schedules; lab-scale success does not always translate to production shear histories and solvent removal profiles, particularly for multilayer stacks where permeability and barrier must be co-optimized [183].

Finally, the standardization of assessment metrics remains a moving target. Cross-study comparisons of “healing efficiency” are obscured by disparate EIS protocols, defect geometries, and exposure regimens; accordingly, recent reviews advocate harmonized test matrices that link electrochemical recovery (EIS/SVET) to visual/rust ratings and mechanical durability over repeated cycles [152]. Addressing these gaps by engineering moisture-tolerant gates, leveraging multi-stimuli triggers, optimizing carrier–binder interfaces, and adopting standardized durability frameworks, will be decisive for qualifying self-healing coatings as reliable, maintainable anticorrosion technologies across marine, aerospace, energy, and civil infrastructure [183].

### 4.2. Self-Healing for Medical Applications

The field is inspired by natural biological processes, such as wound healing, and aims to create synthetic materials that can restore their function and integrity after damage. However, for many medical applications, the healing process needs to be fast and highly efficient. The material must be able to repair damage in a physiologically relevant time frame and restore its original properties, such as mechanical strength and functionality. Furthermore, all the biocompatibility requirements need to be fulfilled to be cost-effective and approved by the corresponding regulatory bodies. In this regard, feedback active coatings are also of great scientific and technological importance in biotechnology and medicine [120,184,185,186].

#### 4.2.1. Hydrogels

**Figure 9:**
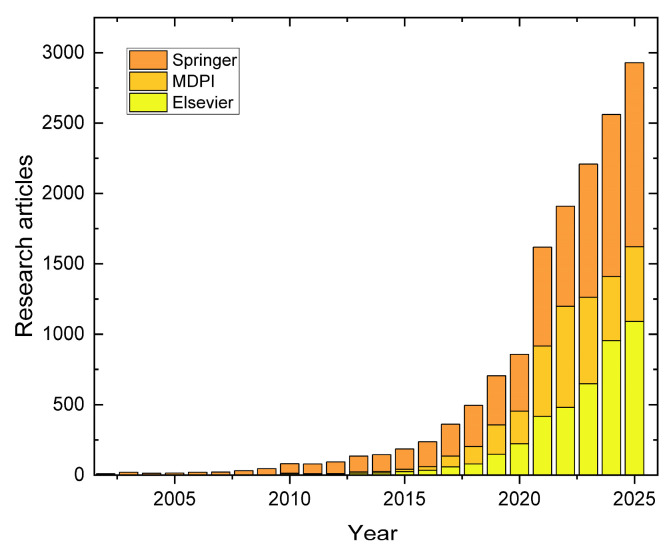
Evolution of publications related to self-healing hydrogels.

Hydrogels are one of the most studied biomedical materials for self-healing applications. Figure 9 shows the time of evolution of the self-healing hydrogel literature until October 2025, according to three different editorial houses. Alongside self-healing materials research, interest in hydrogel-based coatings has been steadily rising (see Figures S1 and S2).

In general, hydrogels are synthesized by using either hydrophilic natural or synthetic polymers, by homo and copolymerization of hydrophilic monomers such as 2-hydroxyethyl methacrylate, and by crosslinking achieved through free radical polymerization, condensation reactions, or coupling reactions such as thiol–ene/yne reactions or alkyne–azide reactions. These materials tend to absorb large quantities of water or water-based fluids. This large amount of water allows the incorporation of drugs, nutriments for cell growth, and high oxygen permeability, rendering them suitable for wound healing and dressing, tissue engineering and regenerative medicine, drug delivery, medical implants and devices, bioelectronics and wearable sensors, and soft robotics [187].

The degree of swelling depends not only on the presence of hydrophilic groups such as OH, NH_2_, COOH, HSO_3_, CONH, etc., but also on the capacity for responding to changes in pH (PAA, PDEAMA), temperature (NIPAA, PEO-PPO), ionic strength, light, etc., rendering intelligent or stimuli-responsive hydrogels. In general, there is a compromise between the amount of water absorbed or retained and their mechanical properties, i.e., the more water, the weaker their mechanical performance. Therefore, serious challenges within the dynamic and mechanically demanding environment of human organs/tissues prevail.

To address numerous issues in the medical field, like the need to restore tissues or morphology after repeated damage while maintaining high toughness, self-repairing hydrogels have been proposed. The ideal self-healing hydrogel should autonomously and rapidly repair under physiological conditions, restoring its mechanical integrity and adapting to various application needs.

**Figure 10:**
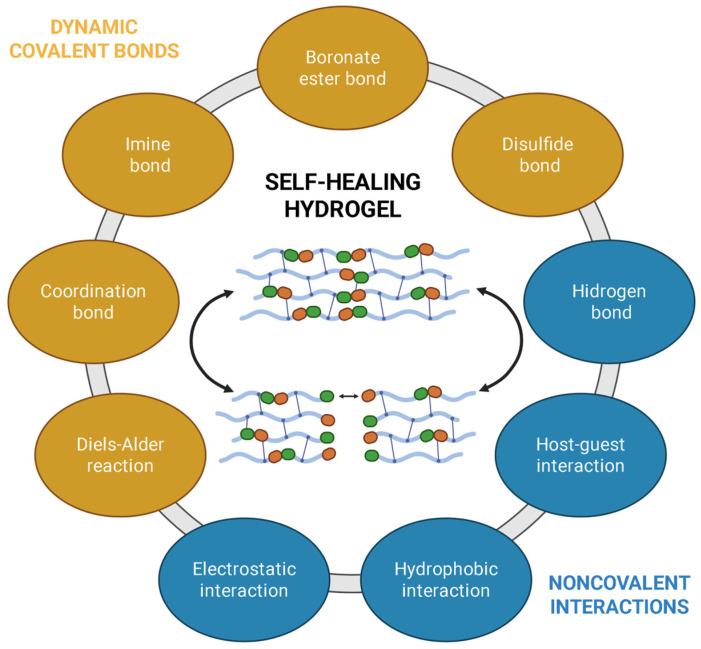
Mechanism involved in self-healing hydrogels. Dynamic covalent bonds (yellow section) and non-covalent interactions (blue section).

As in many self-repairing materials, the underlying mechanism is the same and is based on dynamic covalent bonds that are stable and show slow dynamic equilibrium, and on non-covalent interactions that show fragile and rapid dynamic equilibrium. Some hydrogels are prepared using dynamic bonds that can undergo structural changes in response to a given stimulus by reversibly forming and breaking under one set of conditions and under different conditions, acting as permanent covalent bonds. Dynamic covalent chemistry for the formation of self-healing hydrogel includes imine formation, boronate ester complexation, catechol-iron coordination, Diels–Alder reaction, and disulfide exchange as illustrated in Figure 10 (based on [188]).

##### Dynamic Covalent Bonds

Imines, although less stable than hydrazones, have been exploited by the reaction of aromatic or aliphatic aldehydes with amino-containing polymers like chitosan [189]. As expected, aromatic aldehydes render hydrogels with better mechanical properties and have been proposed for 3D cell culture. By the reaction of hydrazine with aldehydes or ketones or hydroxyalmine to yield hydrazones and oximes, respectively, the imine formation is also possible. Hydrazones are stable under neutral and basic conditions but readily hydrolyze under acidic conditions and exchange in the presence of hydrazides, aldehydes, or ketones. This reaction has been used by Wei et al. (2015) on polysacharide-based hydrogels (alginate-chitosan) for 3D cell encapsulation with 95% of healing efficiency without external stimuli [190].

Boronic acid esters or boronates have also been used in the biomedical field. For example, phenyl boronic acid and cis diol modified polyethylene glycol macronomers formed injectable pH and glucose responsive hydrogels with self-healing properties [191]. A shear-thinning self-healing hydrogel was proposed by Wang et al. (2016) [192] using dynamic covalent diol-benzoxaborole complexation. Here, acrylamide, GAPMA, and MAAmBO were polymerized in the presence of ACVA at pH 9, tris, and DMF. The hydrogel exhibited good mechanical properties and achieved almost 100% recovery after healing in an aqueous solution at physiological pH [192].

Disulfide exchange allows the use of 2-dithiolanes with dithiols to form reversible hydrogels under neutral or mild alkaline conditions that are further controlled by changes in pH, redox potential, and temperature when thiol/dithiolane functionalities are added to F127 [193]. Thiol-functionalized F127 mixed with 1,2-dithiolane-functionalized PEG form injectable hydrogels that exhibited good cytotoxicity with A529 cells [193].

Chelation sometimes recurs for self-healing purposes. Chelation can be described as several coordinate bonds between ligands (organic molecules) and one positively charged transition-metal ion (Fe or Cu). This type of covalent bond possesses high binding energy. Reversible chelation (coordination) between iron and catechol can induce hydrogel formation that is sensitive to changes from acidic to alkaline pH [194]. These bioinspired hydrogels were obtained by 4-arm dopamine-modified PEG crosslinked with F_3_O_4_, rendering unique material mechanics resulting from the supra-molecular cross-link structure dynamics in the gels. In contrast to fluid-like dynamics of transient catechol–Fe^3+^ cross-links, the catechol–Fe_3_O_4_ NP structures provide solid-like yet reversible hydrogel mechanics.

In general, the biomedical applications of the Diels–Alder reaction (a diene that reacts with an alkene or alkyne) are limited because Diels–Alder bonds need a high temperature and a long duration to cleave and reform for self-healing properties. However, in combination with other interaction mechanisms like electrostatic interactions, coordination, imine formation, etc., self-healing hydrogels can be induced.

##### Non-Covalent Interactions

Non-covalent interactions, such as electrostatic interactions, hydrogen bonds, hydrophobic interactions, and supramolecular guest–host interactions, are also used to produce self-healing hydrogels. In Table 8, the different non-covalent interactions are shown by interaction type, focusing on key mechanisms, representative systems, structural features, and performance outcomes.

#### 4.2.2. Applications of Self-Healing Hydrogels

Applications of self-healing hydrogels include wound dressing, drug delivery, tissue engineering surface coating, 3D printing, and soft robotics [209]. Table 9 presents a summary panorama of the different applications of the self-healing hydrogels in the medical area.

### 4.3. Other Biomedical Applications

Self-healable materials, hydrogels, and heat- or electrically conductive materials for self-healable bioelectronics, bioactuators, or actuators (US 201715592313 A), and electronic skin (e-skin) devices can be easily achieved by the incorporation of carbon fibers (US 201514885534 A) or carbon nanostructures such as graphene or carbon nanotubes or carbon nanodots (KR 20230004034 A; KR 20170027008 A; US 201815938016 A; CN 202110790545 A). Self-healing hydrogels based on graphene for wearable strain sensing applications have been recently reviewed [227]. Bioactuators are typically made from a combination of locomotive cells derived from muscle and cardiac tissues, and conductive polymers that can contract and expand. For example, CNTs aligned within GelMA to generate a more native like electrical coupling between encapsulated cardiomyocytes to enable a more coherent bioactuation in physiological conditions [228]. Han et al. (2017) [229] proposed a hydrogel composed of graphene oxide, polydopamine, and polyacrylamide. The hydrogel could be used not only as an adhesive electrode or motion sensor but also as an in vitro cell stimulator and in vivo implantable intramuscular electrode [229].

## 5. Patents and Perspectives for Self-Healing Polymers

Bridging scientific discovery with technological application is crucial. Hence, a compilation of patents on self-healing materials is provided to illustrate ongoing innovation and commercialization efforts in the field; see Table 10.

## 6. Conclusions

This review examined the advances, mechanisms, and practical considerations involved in the design and application of self-healing polymeric systems, with emphasis on coating technologies for corrosion protection and biofunctional interfaces. Self-healing materials are increasingly relevant in engineering, medicine, and biotechnology, where maintaining structural integrity, extending service life, and ensuring reliability under variable environmental or physiological conditions are essential.

Both extrinsic and intrinsic strategies were evaluated. Extrinsic approaches—such as micro- and nanocapsules, microvascular networks, hollow fibers, and inorganic carriers—offer rapid autonomous repair and targeted delivery of healing agents, enabling localized crack closure and corrosion inhibition. However, their limitations include single-use behavior (in capsule-based systems), potential reductions in mechanical performance at high filler loadings, and challenges related to long-term carrier stability and large-scale processing.

Intrinsic self-healing systems based on reversible chemistries, including covalent adaptable networks (CANs) and vitrimers, provide multi-cycle healing, recyclability, malleability, and reprocessability without relying on mobile reservoirs. Dynamic covalent exchanges—such as transesterification, imine metathesis, disulfide exchange, Diels–Alder reactions, and boronic ester interactions—enable repeated restoration of mechanical and barrier properties. Recent advances in epoxy, PDMS, polyurethane, and polyimine vitrimers underscore the potential to tailor activation windows, healing kinetics, and environmental resistance through molecular-level design.

Hybrid approaches that combine polymer matrices with inorganic carriers (mesoporous silica, LDHs, zeolites, halloysite nanotubes, TiO_2_) further expand the functionalities available for protective coatings, enabling pH-, redox-, or mechanical-triggered release alongside passive barrier protection. Nevertheless, the performance of such hybrid systems depends critically on dispersion quality, interfacial compatibilization, cure conditions, and long-term agent retention—all of which remain active challenges, especially for industrial scale-up.

Overall, the comparative analysis presented in this review highlights that no single healing strategy is universally optimal. Capsules provide simple, autonomous repair; microvascular architectures extend healing to multiple cycles; inorganic nanocontainers impart stimulus-responsive functionality; and vitrimer networks offer carrier-free, recyclable, and durable healing. The selection of an appropriate strategy requires balancing mechanical robustness, healing rate, environmental stability, activation requirements, and the demands of the intended application—whether corrosion protection, biomedical interfaces, soft robotics, or high-performance structural materials.

Moving forward, several research directions are essential for advancing self-healing polymer technology toward widespread adoption: (i) the development of standardized performance-testing protocols to allow fair comparison across studies; (ii) integration of extrinsic and intrinsic mechanisms to achieve synergistic multi-modal healing; (iii) exploration of sustainable, bio-based chemistries to enhance environmental compatibility; (iv) long-term stability studies under realistic service conditions, including coupled mechanical, environmental, and electrochemical stresses; and (v) scalable synthesis and processing routes that maintain microstructural fidelity in industrial environments.

By aligning materials chemistry, design principles, and application-driven requirements, self-healing polymer coatings and networks are poised to deliver robust, multifunctional solutions for protective systems and emerging biofunctional technologies.

## Figures and Tables

**Table 1:** Comparative matrix of self-healing paradigms.

Parameter	Extrinsic Self-Healing	Intrinsic Self-Healing
Healing Principle	Release of an encapsulated healing agent.	Inherent reversibility of chemical bonds in the matrix.
Repeatability	Generally limited to a single event; dependent on agent depletion.	Theoretically unlimited; multiple cycles possible.
Typical Stimulus	Autonomous (mechanical damage).	Non-autonomous (heat, light, pH).
Healing Speed	Generally fast.	Variable, can be slower and dependent on chain mobility.
Impact on the Matrix	The presence of containers can act as a defect, affecting mechanical properties.	The matrix design is optimized for repair but may involve a compromise in robustness.
Key Limitation	Depletion of the healing agent.	Trade-off between mechanical properties and healing efficiency.

**Table 2:** Recent advances in polymer–inorganic self-healing composites.

Polymeric System	Application	Reference
Mesoporous SiO_2_ + BTA encapsulated in polymer/sol–gel hybrid	A2024 aluminum alloy; pH-triggered release; sustained EIS during immersion	[48]
Polyethylenimine-wrapped MSNs + inhibitor (pH-sensitive) in coating	Mild steel; active, on-demand release and self-healing behavior	[49]
ZSM-5 zeolite + chitosan (dual pH-responsive nanocontainers) in epoxy	Aluminum alloy; dual self-healing with improved long-term impedance	[50]
ZIF-8 nanocontainers in sol–gel hybrid coating	Aluminum substrates; enhanced long-term protection and self-healing	[51]
NiAl-LDH doped with vanadate (anion-exchange)	AA2024; self-healing via controlled VOx release; ~20× vs. bare Al	[52]
ZnAl-LDH films with intercalated biomacromolecule (MAP)	Al alloys; film-forming inhibitor within LDH; autonomous repair	[53]
Silane nanocomposite (dual self-healing) with inorganic nanocontainers	Aluminum alloy; efficient self-healing and long-term stability	[54]
HNTs (halloysite) as inhibitor carriers in epoxy/acrylate systems	Carbon steel/steel; barrier ↑ and stimulus-responsive release	[55]
HNTs filled self-healing elastomeric nanocomposites (mechanical recovery)	Rubber/structural analogs; crack closing & strength recovery	[45]
Zeolite pigments as eco-safer inhibitor reservoirs in polymers	Metals (general); survey of zeolite-based active pigments	[56]

**Table 3:** Overview of polyurethane vitrimers based on different bond exchange mechanisms.

Type of Bond Exchange	Dynamic Chemistry	Catalyst/Activation	Representative Components	Key Features	Reference
Carbamate (Urethane) Bond Exchange	Transcarbamoylation via associative (alcohol-mediated) or dissociative (alcohol-free) routes	Catalysts: tertiary amines, Lewis acids, organometallics (e.g., DBTL)	PU networks; recycling via DBTL–dichloromethane swelling; reprocessing by compression molding or extrusion	Catalytic exchange promotes recyclability and reprocessability; associative mechanisms favored by alcohols	[101,102,108,109]
Urea Bond Exchange	Hindered urea bonds (HUBs) enable thermally activated urea–urea exchange	DBTDL catalyst; thermal activation	2-(tert-butylamino) ethanol + THDI trimer	Enables stress relaxation, welding, self-healing; retains crosslink density and mechanical strength	[91,103]
Transesterification	Ester–ester exchange in polyester-based PU networks	Catalyst-mediated (unspecified catalyst)	DEM + PTMO diols + MDI + pentaerythritol	Displays rapid stress relaxation, malleability, and healing; suitable for protective films	[101,107]
Imine Bond Exchange	Dynamic imine (C=N) linkages form and exchange reversibly	Catalyst-free; thermally or chemically reversible	Vanillin diol + castor oil + isocyanate systems	Bio-based, recyclable, self-healing, malleable; improved strength with higher vanillin-diol content	[110]
Disulfide Bond Exchange	Disulfide metathesis (S–S bond rearrangement)	UV light or mild heating; catalyst-free for aromatic disulfides	Bis(4-aminophenyl) disulfide; cystamine + bis(cyclic-carbonate) (isocyanate-free PU)	Enables ambient-condition repair, self-healing, welding, solvent resistance	[111]
Boronic Ester Bond Exchange	Boronic ester metathesis and transesterification	Thermal or UV activation; catalyst-free	HTPB + HDI + bis(dioxaborolane) crosslinker	Self-healing, reprocessable, stable PU networks	[112]

**Table 4:** Examples of microvascularized coatings for surface protection.

Type/Architecture	Substrate/Capsule or Vascular Material	Structural Characteristics (Size, Architecture)	Applications	Reference
Microvascular coating	Polyacrylonitrile (PAN) nanofibers/dicyclopentadiene (DCPD) and multiwalled carbon nanotubes	Coaxial electrospinning networks with 500–1100 nm fibers	Protective coatings, aerospace structures	[133]
Wall-less microvascular network	Orthotropic glass-fiber-reinforced polymer/UV-curable resins	Hollow glass fibers (diameter 0.4 mm) filled with epoxy	Structural composites, light repair systems	[134]
Core–shell morphology	Urea–formaldehyde polymer/Epoxy microcapsules micro channels	Capsules ~3–5 µm,	Waterborne wood coatings	[135]
Biomimetic 3D vascular network	Cement-based matrix thermoplastic polymers/sodium silicate	Branched channels, 2 mm inner radius channel	Civil engineering composites, infrastructure	[136]
Sacrificial 3D-printed vascular network	Cementitious composite embedded with Polyvinyl Alcohol PVA tubbing polylactic acid PLA connectors/epoxy resin	Pneumatic hollow channels, elastomeric, diameter ~3 mm. Vascular branched network	Soft robotics, actuators	[137]
Hydrogel scaffolds with oriented, biomimetic capillary-like microvascular networks	alginate-RGD/fibrin + mesenchymal stem cells (MSCs) + human umbilical vein endothelial cells (HUVECs)	Hydrogel matrix with vascular channels ~100–300 µm. Mimic natural endothelial organization in blood vessels	Biomedical hydrogels, wound dressings	[138]

**Table 5:** Performance testing evaluation in microvascular self-healing coating systems.

Architecture/Type	Composition/Material	Healing Performance	Cycles Tested	Applications	Reference
Dual microvascular network	Epoxy-based resin/ductile polymer (PDMS)	Up to 60% fracture toughness recovery	16 cycles	Structural composites, coatings	[130]
Interpenetrating microvascular networks	Epoxy + dual-component adhesive (epoxy monomer, amine-based curing agent)	Efficiency > 50% maintained	≥30 cycles	Automotive panels, load-bearing parts	[156]
Biomimetic branched vascular	Epoxy + continuous fibers/curing agents	89% in tensile strength, 83% in creep rupture time for 7 days	--	Biomimetic composites, civil engineering	[157]
Fiber- laminated composite	glass/carbon fiber and thermoplastic, coupled with resistive heater interlayers	Full fracture recovery. Tensile stress–strain, tested at temperatures > 110 ^∘^C	100	Wind-turbine blades aerosurfaces	[158]
Bilayer constructs	Polyethylene glycol and prednisolone	>90% recovery from fracture	3	Flexible electronics, -e-skin	[159]
Layer-specific nanofiber membranes and epoxy blockage layer	Polycaprolactone carbon nanotubes/epoxy	Self-healing anticorrosion efficiency of 96.32%	--	Anticorrosion local microdeffects	[151]
Composite (soft + hard) hybrid structure	Oleogel microspheres and waterborne epoxy matrix	80% reduction in friction coefficient, 63% reduction in wear volume, adhesion strength of 96%	More than 110 days	Extremely harsh environments	[160]

**Table 6:** Representative self-healing coating systems for corrosion protection.

Systems	Self-Healing Results	Reference
MCM-41 (MSN) nanocontainers gated by PEI/PSS + triazinyl inhibitor	Q235 steel; alkaline-triggered release; ≈10^3^, increase in low-frequency impedance after 30 days; autonomous restoration at defects	[58]
PEI-wrapped MSNs + BTA	Mild steel; high pH-sensitivity; on-demand release; notable EIS gains after cycling	[49]
ZIF-8@SiO_2_ (MOF–silica core–shell) in sol–gel/epoxy hybrids	Al; MOF core provides pH-triggered cargo; silica shell improves compatibility/leakage control; multi-cycle EIS recovery and scratch healing	[170]
HNTs@MBT/MBI/BTA in PU/acrylic	Copper & stainless steel; inhibitor storage in lumen; durable scratch-healing and active protection	[62]
Zeolite ZSM-5 + chitosan (dual pH-responsive) in epoxy	Aluminum alloys; dual container/gate prolongs self-healing window; improved long-term impedance	[50]
Zeolite pigments	Metals (general); eco-safer inhibitor reservoirs; ion-exchange-based self-healing	[56]
LDH/LHS intercalated with MoO_4_^2−^ in epoxy	Carbon steel; anion-exchange “smart” release; strong self-healing validated by EIS/SVET; suppressed pit growth	[56]
TiO_2_ nanocapsules@BTA in waterborne PU/GO	Steels/Al; dual self-healing (inhibitor + barrier); multi-cycle recovery	[64]
CeO_2_@SiO_2_ with phenanthroline in PU	Al alloys; hydrophobic, UV-resistant, self-healing with corrosion detection	[44]
NaY zeolite co-loaded with Ce^3+^ and DEDTC in epoxy	AA2024-T3; ion-exchange-driven dosing; combined cathodic passivation and anionic inhibition; active defect protection	[171]

**Table 7:** Overview of anticorrosive self-healing materials and applications.

Sector	Key Conditions	Self-Healing Strategy	Nanocontainer or System Used	Performance	References
Automotive and Heavy Transport	Wet–dry cycles, de-icing salts, stone-chip damage, galvanic coupling at fasteners	pH-responsive inhibitor release; intrinsic vitrimer healing	MSNs (≈2–8 nm) loaded with benzotriazole (BTA); Zn–BTA “stoppers”; PEI-wrapped MSNs; epoxy vitrimers	Sustained impedance	[48,49,59,89]
Marine and Offshore Environments	Salt spray, biofouling, immersion, chloride fluxes	Anion-exchange and pH-responsive inhibitor release	Zn–LDH intercalated with molybdate (MoO_4_^2−^); LDH (vanadate, phosphate); zeolite pigments (ZSM-5 + chitosan); TiO_2_ nanocapsules@BTA in PU/GO	Defect-selective inhibition; sustained high	[91,178,179]
Aerospace Structures and Lightweight Alloys	Localized corrosion at fasteners and fretting scars; cyclic mechanical stress	pH-triggered inhibitor release; hybrid carrier systems; vitrimer-based resealing	MSN–BTA coatings; MOF@SiO_2_ core–shell (BTA–ZIF-8@SiO_2_); NaY zeolite co-loaded with Ce^3+^ and DEDTC; epoxy vitrimers with sol–gel primers	Sustained high	[48,91]
Energy, Pipelines, and Civil Infrastructure	Cyclic wetting, fluctuating pH/salinity, stray currents, soil exposure	Gradual and defect-localized inhibitor release; in situ crosslinking (“chemical closure”)	HNTs and MSNs; BTA@PANI–HNTs; TiO_2_ nanotubes + MSNs co-releasing epoxy prepolymer and amine hardener; multifunctional colloidosomes	Up to 4-order	[63,86,180,181]
Electronics and Protective Housing	Condensation and thermal cycling in sealed enclosures	Intrinsic vitrimer self-healing; thermal and chemical bond exchange	PDMS-based vitrimers (boronic ester and silyl ether types) layered over inhibitor-bearing primers	Hydrophobic transparent coatings; repeatable scratch recovery under mild heating; reprocessability; wetting resistance	[91,182]

**Table 8:** Non-covalent interactions in self-healing hydrogels.

Interaction Type	Mechanism	Representative Systems	Reported Self-Healing/Performance	Reference
Hydrogen bonds	Dynamic H-bonding between donors/acceptors; stabilization via multiple/quadruple H-bonds (e.g., UPy), hydrophobic pockets	UPy-rich supramolecular networks; catechol/phenylboronate dynamic bonds; DNA/polypeptide–polymer H-bonded matrices	Fast room-temp healing; tissue adhesion; improved toughness via complementary/multiple H-bonds	[127,195,196]
Hydrophobic interactions	Hydrophobic association (HA) domains; micelles/liposomes; cholesterol-decorated chains; dual networks (hydrophobic + ionic)	Micellar/HA hydrogels for drug delivery; cholesterol-engineered hydrophobic surfaces	Toughness + rapid recovery from reversible HA; wet-surface adhesion without toxic crosslinkers; micellar gels for controlled release	[197,198,199]
Supramolecular guest–host	Cyclodextrin (α/β/γ) or CB(8) hosts with adamantane, ferrocene, carborane, etc.; sometimes boosted by π–π stacking	β-CD–guest hyaluronic acid networks; CB–guest dextran/PAA systems; CD-based hydrogels for TE/regen med	Shear-thinning, injectability; full/rapid G′ recovery; modularity for bioactive delivery	[200,201,202]
Electrostatic interactions (polyelectrolytes/zwitterions)	Reversible ionic crosslinks (PECs) between polycations/polyanions; zwitterionic hydration shells; pH/ionic strength tunability	PEC hydrogels (e.g., PAAm/PAA–Fe^3+^, chitosan–sulfated polysaccharides); zwitterionic (carboxybetaine/sulfobetaine) networks	High toughness, anti-fatigue, anti-freezing; fast modulus recovery; antifouling, conductive, stretchable	[155,203,204,205,206]
Interpenetrated/double networks (DN/IPN)	Strong + weak network synergy (e.g., covalent PAAm + physical/ionic alginate or entanglement-dominated DN)	Highly entangled DN without dissipative sacrificial bonds; DN gel polymer electrolytes	High toughness with low hysteresis; rapid recovery; stretchable, self-healing electrolytes	[207,208]

**Table 9:** Self-healing hydrogels in medicine.

Application	Mechanisms Enabling Self-Healing	Clinically Performance	Representative System	Reference
Drug delivery and cell encapsulation	Host–guest (β-CD/adamantane), dynamic imine/Schiff base, boronate ester; shear-thinning for injection	Local retention, on-demand release (incl. RNA), cytocompatibility, in situ gelling	HA–β-CD/adamantane injectables for RNA/biologics; aldehyde–amine chitosan/PEG carriers	[210,211]
Steroid-drug self-assemblies (anti-inflammatory/osteo)	Ionic coordination (e.g., Ca^2+^) + supramolecular UPy H-bonding; reversible physical networks	Shear-thinning injectability, sustained Dex release, osteogenic support	Dex-loaded, self-healing UPy/gelatin–ZnHAp injectable microspheres for bone	[212,213]
Bone/dental repair	Dual crosslinking (ionic chelation with Ca^2+^/Mg^2+^ + UV/covalent), dynamic HA networks, bioactive glass interactions	Defect filling, cell spreading/osteogenesis, mechanical integrity, injectability	PEG-thiol + bioactive glass; dynamic HA composites; bioinspired injectable systems	[214,215]
Cartilage repair	Dynamic covalent (acylhydrazone, Schiff), Diels–Alder, supramolecular (β-CD/guest), double networks; sometimes SM (shape-memory)	Adhesion to cartilage, high toughness, self-repair under load, ECM restoration	Dynamic HA hydrogels for articular cartilage; AHA/CHA systems restoring ECM	[216,217]
Skin substitutes/wound dressings	Catechol/boronate dynamic bonds, Schiff base (aldehyde–amine), electroconductive fillers (polyaniline, PDA, carbon)	Tissue adhesion, rapid self-healing, antibacterial/anti-oxidative action, sensor integration	Conductive chitosan-PANI, PDA-based dressings; multifunctional adhesive dressings	[218,219]
Cardiac tissue	Host–guest HA networks (β-CD/adamantane) with secondary covalent crosslinking; conductive components for coupling	Injectability, retention in myocardium, improved electromechanics, angiogenesis	Self-healing cardiac hydrogels and conductive patches/scaffolds	[220,221]
Nervous system	Low-modulus chitosan/alginate imine gels; boronate esters; conductive fillers (graphene) with dynamic links	Modulus match to neural tissue, neurite outgrowth, minimally invasive injection, vascularization strategies	Injectable chitosan–oxidized alginate; boronate-ester sacrificial constructs; conductive neural scaffolds	[222,223]
Shape-memory and multi-responsive systems	Dynamic covalent (boronate, imine), supramolecular (β-CD/guest, UPy), metal–ligand; IPNs; 4D-printing formulations; water-soluble polymer blends with shape memory effect via hydrogen bonding	Programmable shape fixing/recovery near body T, repeated healing cycles, multi-trigger actuation (pH/ion/UV/T), water-induced shape memory with nearly 100% recovery	4D-printed self-healing SM hydrogels; multi-responsive IPNs for actuation + healing, PVA/p-coumaric acid modified chitosan	[120,224,225,226]

**Table 10:** Comparative summary of self-healing polymer and material patents.

Application Domain ^(b)^	Representative Mechanisms/Material Types ^(a)^	Patent Codes	Countries ^(c)^	Reference
1. Anti-corrosion and protective coatings	Microcapsule-based epoxy systems, double-layer (PU/PUF) capsules with diisocyanate core, metallic microcapsules, alkyd and epoxy–amine healing resins, corrosion-responsive capsule release	US 201113083819 A; US 201414303494 A; US 202017130402 A; US 201916597245 A	USA, South Korea, PCT/WO	[54,230,231,232,233,234]
2. Structural composites and aerospace	Dual-capsule FROMP p(DCPD); vascular microchannels and optical fibers; SHM-triggered capsule rupture; magnetic or heat-triggered capsules; catalyst-decorated polymeric microcapsules	US 2024/0021718 W; US 201213721801 A; US 201414309484 A; US 201715612256 A; US 201715618589 A; EP 16382598 A; US 202318467968 A	USA, Europe, PCT/WO	[144,145,177,235]
3. Capsule-based self-healing in polymer matrices	Orthogonally graftable polymer shells; multi-shell micro/nano-capsules; ENB-filled ROMP capsules; nanotube end-capped reservoirs	US 201213482366 A; KR 20110116295 A; KR 20120024281 A; US 201815938016 A	USA, South Korea	[40,236]
4. Elastomers and soft materials	Reversibly crosslinked siloxane elastomers; PDMS/B_2_O_3_ elastomers; metal-ligand elastomers; conductive PEDOT:PSS–silicone hybrids	CA 3165584 A; US 202218559401 A; US 201213686150 A; US 202318847485 A	Canada, USA	[237]
5. Electronics and flexible devices	UV-responsive disulfide-exchange networks; OLED flexible glass with self-healing layer; transparent conductive elastomers; dynamic covalent polyurethane	US 202418610294 A; US 201916531447 A; US 202318847485 A; KR 20220114363 A; KR 20180009843 A	USA, South Korea	[238,239,240,241]
6. Energy storage (batteries & supercapacitors)	Gel polymer electrolytes (UPyMA, PU-Li^+^ systems); self-healing positive electrodes; supramolecular polymeric electrodes	CN 202411662896 A; CN 202411754197 A; US 201514639552 A	China, USA	[242,243,244]
7. Medical, biomedical & pharmaceutical systems	Porous self-healing matrices for macromolecule encapsulation; pH-modulated polyelectrolyte complexes; silica-shell dental composites; neural interfaces; diabetic-wound hydrogels; chitosan–aldehyde gels	US 2011/0021166 W; US 201916532120 A; US 201514952492 A; US 202016902040 A; CN 202411551605 A; CN 202211739917 A; CN 202010702427 A	USA, China	[245]
8. Natural polymer & hydrogel systems	PVA–metal ion coordination (Zn^2+^); phenol-modified chitosan–aldehyde; aldehyde-chitosan–alginate–Cu/Au; mussel-inspired injectable hydrogels; supramolecular polysaccharide networks	US 2020/0054077 W; CN 202211739917 A; CN 202010702427 A; US 201615389918 A	USA, China	[246]
9. Construction, infrastructure & civil materials	Polymer-modified cements; anti-CO_2_-corrosion core–shell microcapsules; roofing adhesives; asphalt with microcapsule agents; self-healing paints	US 202117453297 A; CN 2023141836 W; US 201816644889 A; MX 2018000719 A; KR 20140078543 A	USA, China, Mexico, South Korea	[247,248,249,250]
10. Textiles, inflatables & cables	Fabric laminates with internal/external healing layers; self-sealing water-swellable cable compositions	US 202117471504 A; US 92343601 A	USA	[251,252]
11. Water treatment & anti-fouling	Self-healing reverse-osmosis and polymeric membranes using nano-healing agents and anti-fouling polymers	CN 202411645168 A; CN 201710242909 A	China	[253]
12. Environmental & extreme-condition coatings	Ice-resistant self-healing silicone–hydrophobic composites; corrosion-resistant SH coatings	CN 202210266797 A; US 202217883306 A	China, USA	[254]
13. Optical, luminescent & sensing materials	Photo-luminescent supramolecular polymers; TADF-based hydrogen-bonded thermoplastic networks for OLEDs	KR 20210086845 A; CN 202411533252 A	South Korea, China	[255]
14. Automotive sector	PU micro/nano-capsule coatings; self-healing joining structures with rivets; omniphobic reversible coatings; disulfide PU polymers	US 201715408859 A; US 201514955664 A; US 202017419778 A; KR 20220114363 A	USA, South Korea	[231,256]
15. Historical foundational patents	Polyurethane-based laminates and adhesives introducing the concept of “self-healing” surface reforming	US 4623592 A; EP 0190700 A2	USA, Europe	[257,258,259,260]

^(a)^ Mechanism spectrum: includes microcapsule rupture, dynamic covalent bonds (disulfide, Diels–Alder, coumarin), supramolecular H-bonding, metal–ligand coordination, reversible crosslinking, and vascular microchannel delivery. ^(b)^ Application breadth: spans coatings, elastomers, electronics, biomedical devices, batteries, and construction, demonstrating the convergence of self-healing chemistry across industries. ^(c)^ Geographical trends: USA and China dominate in patent filings (especially post-2018); South Korea leads in multifunctional polyurethane and asphalt systems; Europe/Canada/Mexico contribute through early development and niche uses (siloxanes, paints).

## Data Availability

No new data were created or analyzed in this study. Data sharing is not applicable to this article.

## Supplementary Materials

The following supporting information can be downloaded at: https://www.mdpi.com/article/10.3390/polym17233154/s1, Figure S1: PRISMA flow diagram for systematic review of self-healing polymer materials; Figure S2: Keyword co-occurrence network of the literature dataset generated with VOSviewer, version 1.6.20.
