# Supplementary material for: Self-Healing Polymer-Based Coatings: Mechanisms and Applications Across Protective and Biofunctional Interfaces
Source: Polymers (Basel). 2025 Nov 27;17(23):3154. doi: 10.3390/polym17233154 (PMC12694030; doi:10.3390/polym17233154)

Supplementary Information

S1. PRISMA Methodology

To ensure reproducible selection of the literature, the database search and screening process was structured according to the PRISMA guidelines. The literature search was conducted in Scopus using advanced queries designed in accordance with the PRISMA methodology. The specific search strings used for each application field were as follows:

- Corrosion applications. TITLE (("self-healing" OR "self healing") W/1 (coating*)) AND TITLE-ABS-KEY (corrosion OR anticorrosion OR "anti-corrosion") AND PUBYEAR > 2015 AND PUBYEAR < 2027 AND (LIMIT-TO (DOCTYPE , "ar") OR LIMIT-TO (DOCTYPE , "re")) AND (LIMIT-TO (SUBJAREA , "MATE") OR LIMIT-TO (SUBJAREA , "CHEM") OR LIMIT-TO (SUBJAREA , "CENG")) AND (LIMIT-TO (LANGUAGE , "English"))
- Biomedical applications. TITLE (("self-healing" OR "self healing") W/1 (hydrogel* OR "micellar hydrogel" OR "double-network hydrogel")) AND TITLE-ABS-KEY ("tissue engineering" OR "tissue regeneration" OR "biomedical") AND PUBYEAR > 2015 AND PUBYEAR < 2027 AND (LIMIT-TO (DOCTYPE , "ar") OR LIMIT-TO (DOCTYPE , "re")) AND (LIMIT-TO (SUBJAREA , "MATE") OR LIMIT-TO (SUBJAREA , "BIOC") OR LIMIT-TO (SUBJAREA , "MEDI") OR LIMIT-TO (SUBJAREA , "IMMU"))

The PRISMA flow diagram showed in **Figure S1** summarizes the progressive reduction of records from identification to final inclusion based on the eligibility criteria defined in this review.

Figure S1. PRISMA flow diagram for systematic review of self-healing polymer materials

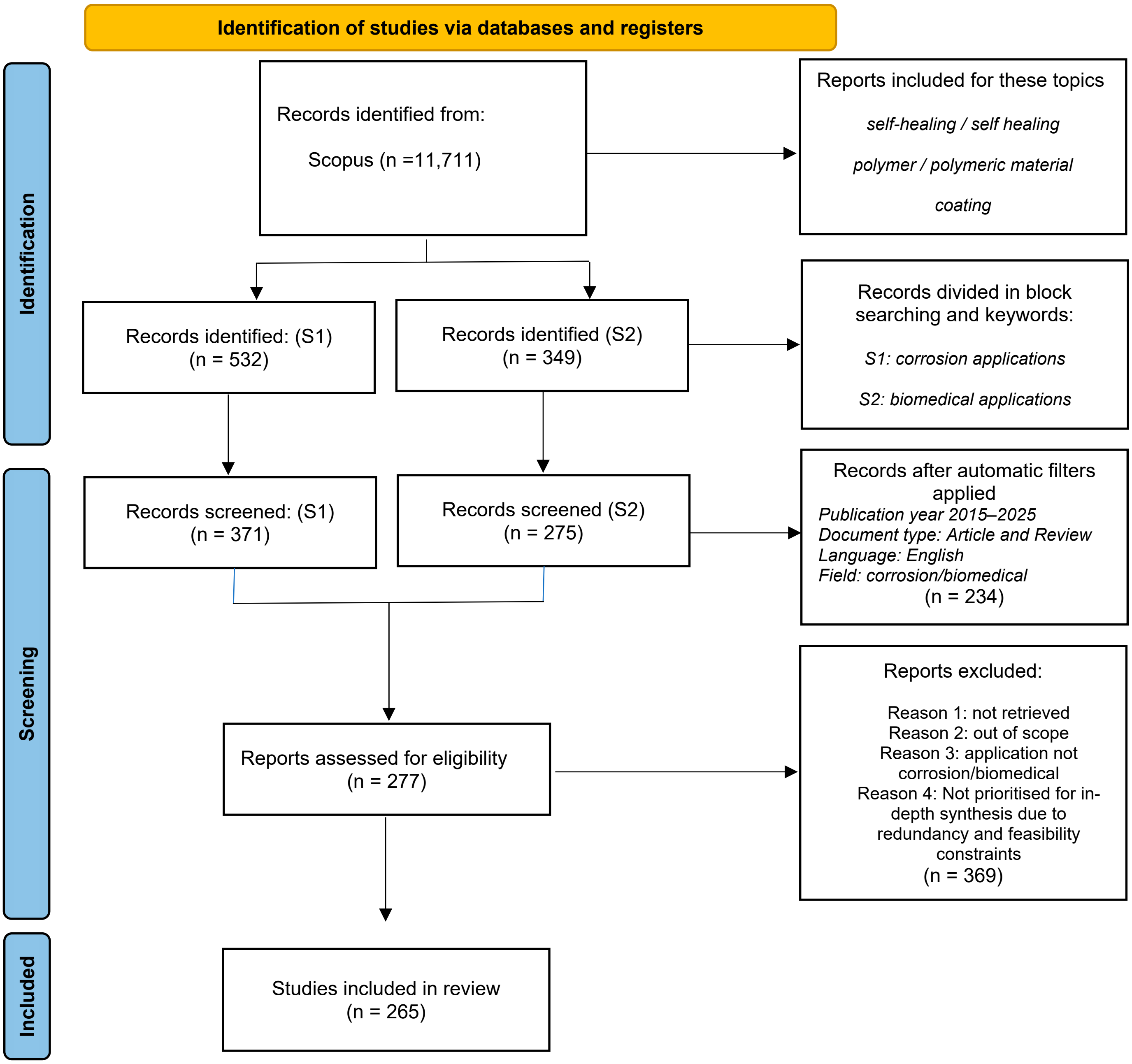

S2. VOSviewer

Additionally, a bibliometric keyword co-occurrence analysis was carried out with VOSviewer software using the final dataset. **Figure S2** shows the resulting network map which highlights the main research clusters and emerging themes related to self-healing polymer-based coatings across different application fields.

Figure S2. Keyword co-occurrence network of the literature dataset generated with VOSviewer.

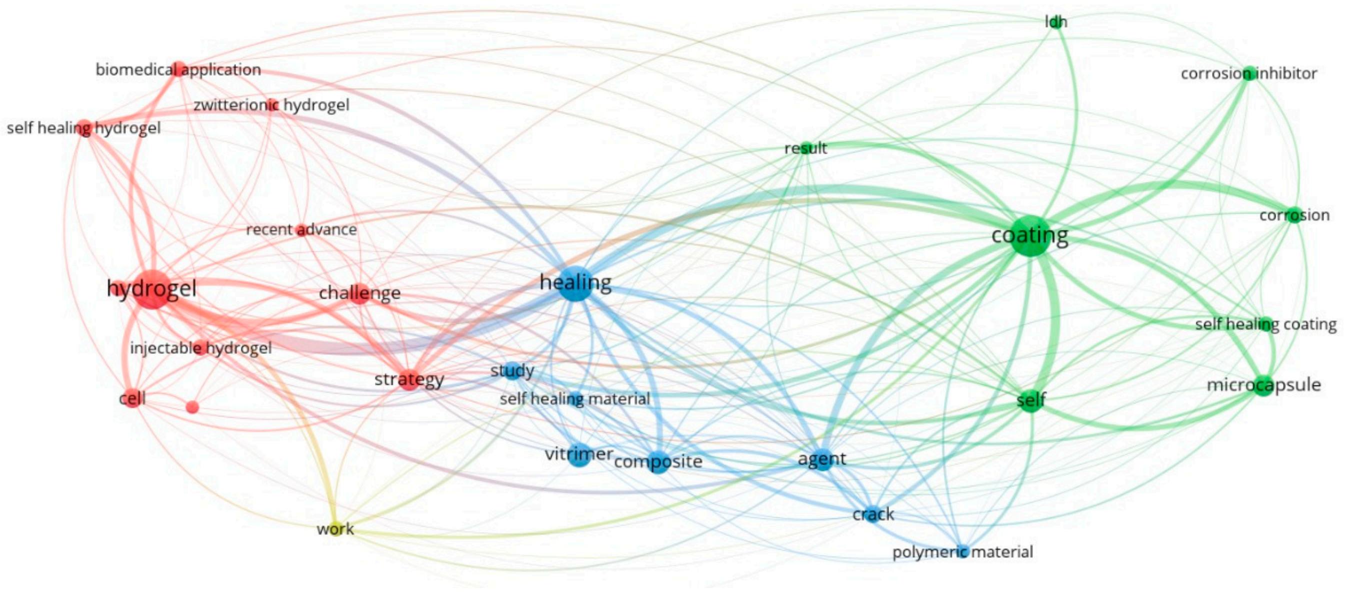

Supplement: Supplementary file 1 [file polymers-17-03154-s001.zip › polymers-3986053-supplementary.pdf]
